# A Distributed 4-Port Hilbert Slot MIMO Antenna with Full-Body Phantom Validation for mmWave Wearable Telemetry

**DOI:** 10.3390/s26175503

**Published:** 2026-08-30

**Authors:** Sahar Saleh, Tale Saeidi, Nick Timmons

**Affiliations:** 1 WiSAR Lab, Atlantic Technological University (ATU), F92 YY97 Letterkenny, Co. Donegal, Ireland; tale.saeidi@atu.ie (T.S.); nick.timmons@atu.ie (N.T.); 2 Department of Electronics and Communications Engineering, Faculty of Engineering, University of Aden, Aden 5243, Yemen; 3 Electrical and Electronics Engineering Department, Faculty of Engineering and Natural, Sciences, Istinye University, 34326 Istanbul, Turkey

**Keywords:** Hilbert-slot MIMO antenna, super-wideband antenna, 5G FR2 mmWave, wearable medical telemetry, body-centric communication, envelope correlation coefficient, diversity gain, specific absorption rate, link-margin analysis

## Abstract

This paper proposes a compact four-port Hilbert-slot MIMO antenna (HSMA)for 5G FR2 mmWave wearable medical telemetry and body-centric communication. The antenna is realized on a semi-flexible Rogers RT/Duroid 5880 substrate with an overall footprint of 12.32 × 12.32 × 0.508 mm^3^. The proposed work develops a compact Hilbert-slot radiator into a four-port 2 × 2 MIMO configuration to enhance port diversity, inter-port isolation, and body-centric link robustness. The simulated four-port HSMA provides super-wideband impedance matching from 24.05 to 62.64 GHz, while the fabricated prototype achieves measured matching from 24.71 to 63.81 GHz, corresponding to a 39.1 GHz measured bandwidth. The measured inter-port transmission coefficients remain mostly below −20 dB across the main operating band, confirming suitable MIMO isolation within the compact layout. The proposed HSMA also demonstrates practical radiation and diversity performance over the highlighted 24–64 GHz band. The measured realized gain reaches 7.52 dBi, and the measured radiation efficiency remains between 51.43% and 76.23%. The calculated measured MIMO diversity parameters show ECC values below 0.0175 and DG values above 9.998 dB, confirming weak correlation between ports and near-ideal diversity behavior. To assess wearable suitability, the antenna is further investigated under bending and realistic body-loading conditions using the CST Gustav voxel human model. In addition, orientation-based body placements are considered to evaluate telemetry paths between distributed body-worn nodes. CST-simulated S_21_ parameters are then used to calculate received power and link margin, providing a link-level assessment of the proposed wearable telemetry system. The results show that the proposed compact four-port HSMA combines continuous super-wideband operation, good MIMO isolation, low ECC, high DG, SAR compliance, bending robustness, and body-centric link feasibility, making it a promising candidate for 5G FR2 wearable biomedical telemetry, WBAN, and future mmWave body-centric communication systems.

## 1. Introduction

Wearable medical telemetry systems are increasingly required to support continuous monitoring, high-data-rate physiological sensing, and reliable communication between body-worn devices. As wearable platforms become smaller and more data-intensive, conventional low-frequency links may not always provide sufficient bandwidth for emerging applications such as real-time health monitoring, high-resolution sensing, and short-range body-centric data exchange. The 5G millimetre-wave (mmWave) frequency range, particularly the Frequency Range 2 (FR2) spectrum, offers large available bandwidth and enables compact antenna dimensions, making it attractive for next-generation wearable and biomedical communication systems [1].

Despite these advantages, mmWave wearable communication remains challenging. At high frequencies, electromagnetic waves experience strong free-space attenuation, body-induced absorption, shadowing, and sensitivity to antenna orientation [2,3]. These effects become more severe when antennas are placed close to lossy human tissues or distributed across different body regions, where body proximity can affect impedance matching, radiation efficiency, and safety-related parameters such as SAR [4,5]. In addition, compact multi-element wearable antennas must maintain good impedance matching, high isolation, low correlation, and stable radiation behavior within a limited physical area [6]. Therefore, the design of wearable mmWave antennas should not only consider conventional antenna parameters, but also the reliability of the communication link under realistic body-centric conditions [7].

Fractal antennas have attracted considerable attention for compact and wideband wireless systems because their self-similar and space-filling geometries can increase the effective electrical length without significantly enlarging the physical antenna size. This feature is particularly useful at mmWave frequencies, where wearable devices require low-profile radiators that can be integrated into small biomedical nodes while maintaining acceptable bandwidth and radiation performance. Among different fractal configurations, Hilbert-type geometries are especially attractive because their folded current path supports compactness, multi-resonant behavior, and wideband operation using a relatively simple planar structure. These properties make Hilbert fractal antennas suitable candidates for wearable mmWave systems where size, bandwidth, and integration constraints must be addressed simultaneously [8].

Although compact fractal antennas can provide wide bandwidth and miniaturization, a single radiating element may not be sufficient for reliable wearable telemetry in realistic body-centric environments. At mmWave frequencies, the human body introduces strong absorption, shadowing, detuning, and orientation-dependent path loss [2,3]. Multiple-input multiple-output (MIMO) technology can help address these limitations by improving diversity performance, reducing link instability, and increasing the probability of maintaining a reliable communication path between body-worn nodes. Therefore, combining a compact Hilbert fractal radiator within a MIMO configuration provides a promising route for achieving both antenna miniaturization and robust wearable telemetry performance. MIMO antenna technology provides an effective approach for improving wearable link reliability, diversity performance, and robustness against fading and body blockage. By using multiple antenna ports or distributed antenna elements, MIMO systems can reduce the effect of orientation mismatch and allow the communication system to select a stronger propagation path between body-worn nodes [6,7]. This is particularly important for wearable medical telemetry, where uninterrupted data transmission may be required during movement, posture variation, or partial body shadowing. For this reason, recent studies have increasingly investigated flexible and compact mmWave MIMO antennas for 5G wearable applications [4,5,6,7].

Several recent works have reported four-port or multi-port mmWave MIMO antennas for wearable and biomedical applications. For example, flexible four-port designs have been demonstrated for 28/38 GHz and tri-band FR2 operation, with reported evaluations of isolation, envelope correlation coefficient (ECC), diversity gain (DG), channel capacity loss (CCL), total active reflection coefficient (TARC), bending behavior, and specific absorption rate (SAR) [6,7]. Other studies have extended the analysis to linear MIMO arrays and biomedical telemetry link-budget evaluation [7]. These works confirm the growing importance of mmWave MIMO antennas for wearable systems; however, many reported designs are limited to narrow, dual-band, tri-band, or quad-band operation rather than continuous super-wideband coverage [6,7].

In parallel with the development of wearable MIMO antennas, fractal-based configurations have also been widely investigated to improve compactness, bandwidth, and inter-element isolation. For example, a compact multiband 4-element MIMO antenna using Sierpiński fractal radiators and a Hilbert fractal Composite Right/Left-Handed (CRLH) band-stop filter was reported in [9], where the fractal/metamaterial loading was used to suppress surface-wave coupling and improve near-field isolation. At mmWave frequencies, other fractal and array-based designs have also been explored to enhance bandwidth and gain while maintaining compact dimensions. A 4-port orthogonally arranged cross-fractal MIMO antenna with metamaterial resonators achieved wideband operation from 15.97 to 45.12 GHz with high isolation [10], while circular, Sierpiński, Minkowski–Sierpiński, Koch, and hybrid Minkowski–Koch fractal geometries have been used to realize compact multiband or wideband antennas for 5G mmWave, WBAN, IoT, and telemedicine applications.

Several fractal-based array and MIMO configurations have been investigated to improve compactness, bandwidth, and isolation for 5G mmWave applications. Mondir et al. [11] proposed a compact 1 × 3 patch antenna array operating from 27 to 30.86 GHz, where bandwidth enhancement and miniaturization were achieved by integrating a complementary infinite split-ring resonator (CI-SRR) on the radiator and a Hilbert fractal structure on the ground plane. To reduce mutual coupling, CRLH structures were incorporated on the backside of the antenna. Similarly, a hybrid fractal MIMO antenna combining Minkowski and Koch curves on a half-octagonal patch was reported for wideband 5G and UWB applications [12], where a tapered feed and a modified ground were used to improve bandwidth and isolation. Other fractal configurations have also been explored, including modified rectangular-slot fractal antennas for 5G mmWave applications [13], spherical-shaped fractal-slot MIMO antennas for multi-band 5G operation [14], and 2 × 2 Minkowski fractal MIMO antennas for 5G and IoT mobile terminals [15].

More recent studies further confirm the growing interest in compact fractal and flexible MIMO antennas for mmWave and wearable systems. A compact fractal-based super-wideband mmWave MIMO antenna using four Koch fractal monopole elements was reported for 5G NR and prospective 6G services, achieving wide mmWave operation, high isolation, and favorable diversity performance [16]. A natural fabric-based wearable fractal MIMO antenna was also proposed for 5G and next-generation wireless systems, demonstrating flexible operation, high isolation, low ECC, and high diversity gain [17]. In addition, recent four-port mmWave MIMO antennas have been developed for 5G FR2 and wearable applications, including tri-band, dual-band, and quad-band designs with evaluations of isolation, ECC, DG, CCL, TARC, SAR, bending behavior, and link-budget performance [6,7]. These works demonstrate the importance of MIMO techniques for improving diversity and reliability in wearable mmWave systems.

However, despite these advances, most reported designs are still limited to discrete dual-band, tri-band, quad-band, or sub-band operation rather than continuous super-wideband coverage across the main FR2 spectrum. Moreover, many studies focus mainly on antenna-level metrics, such as reflection coefficient, isolation, radiation pattern, ECC, DG, CCL, TARC, and SAR, while fewer works evaluate realistic body-centric telemetry links using full-body phantom placements and CST-simulated transmission coefficients. In practical wearable medical systems, antennas may be positioned on different body regions, such as the head, chest, wrist, leg, or shoulder, where link quality can vary significantly because of tissue loading, body curvature, shadowing, posture variation, and relative antenna alignment. Although simplified bending or local phantom models are useful for initial validation, they may not fully represent practical propagation paths between distributed body-worn nodes. Therefore, a more realistic assessment should combine MIMO antenna metrics with full-body phantom simulations and link-level quantities, such as received power and link margin.

To address these limitations, this paper proposes a compact super-wideband four-port Hilbert-slot MIMO antenna for mmWave wearable medical telemetry. The proposed HSMA operates across the main 5G FR2 mmWave region while maintaining compact dimensions, wide impedance matching, and low inter-port coupling. The Hilbert-slot geometry provides a compact radiating structure by increasing the effective current path within a limited physical area, while the four-port 2 × 2 MIMO configuration supports port diversity and improves body-centric link robustness.

The proposed antenna is assessed using free-space, bending, layered phantom, and full-body voxel phantom scenarios. Different body placements are considered to evaluate impedance stability, mutual coupling, radiation behavior, SAR, and body-induced detuning under practical wearable conditions. In addition, CST-simulated S_21_ parameters are used to evaluate representative body-centric telemetry links, where the calculated received power and link margin provide a practical assessment of the antenna’s suitability for short-range and mid-range wearable medical monitoring applications.

The main contributions of this work are summarized as follows:

1. A compact four-port Hilbert-slot MIMO antenna (HSMA) is proposed for super-wideband mmWave wearable telemetry over the 24–64 GHz region.
2. The 2 × 2 MIMO layout provides a compact size, good inter-port isolation, and low mutual coupling.
3. The antenna is evaluated under free-space, bending and CST Gustav full-body phantom conditions.
4. MIMO diversity is verified using ECC and DG for the four port combinations.
5. CST-simulated S_21_ link analysis is used to assess received power and link margin for body-centric telemetry paths.

The remainder of this paper is organized as follows. Section 2 presents the antenna design and simulation methodology, including the development of the compact four-port HSMA from the single element, parametric optimization, bending analysis, body-loading evaluation, SAR calculation, and CST-simulated telemetry link analysis. Section 3 discusses the simulated and measured results, including impedance matching, inter-port coupling, realized gain, radiation efficiency, radiation patterns, ECC, DG, and comparison with recent MIMO antennas. Finally, Section 4 concludes the paper. All full-wave simulations are carried out using CST Microwave Studio based on the finite integration technique (FIT).

## 2. Antenna Design and Simulation Methodology

This section presents the design and simulation methodology of the proposed compact super-wideband four-port HSMA for wearable mmWave telemetry applications. The antenna is developed by arranging four compact Hilbert-slot radiating elements in a 2 × 2 MIMO configuration to support port diversity, inter-port isolation, and body-centric link reliability. The design process includes the optimization of the Hilbert-slot element, the formation of the four-port MIMO layout, parametric analysis, bending evaluation, body-loading assessment, SAR calculation, and CST-simulated S_21_-based telemetry link analysis. Section 2.1 describes the design evolution and optimization of the four-port HSMA starting from the single element. Section 2.2 presents the bending and on-body performance. Section 2.3 presents the SAR analysis to verify electromagnetic safety. Finally, Section 2.4 investigates the body-centric telemetry link performance using CST-simulated S_21_ parameters, received power, and link margin.

### 2.1. Design Evolution from Single Element to Four-Port MIMO Antenna

The proposed four-port HSMA is developed in two main steps. First, a compact Hilbert-slot radiating element is optimized to obtain super-wideband operation across the 5G FR2 mmWave spectrum. Second, the optimized element is arranged into a four-port MIMO configuration to improve link robustness and enable spatial diversity for wearable telemetry applications. This design strategy allows the compactness and wideband behavior of the Hilbert-slot radiator to be retained while extending its functionality from a single antenna element to a multi-port body-centric communication system.

The Hilbert-slot geometry is used as the elementary radiator because its folded current path increases the effective electrical length within a limited physical area. This property supports compactness and wideband behavior, which are important for wearable mmWave antennas.

**Figure 1:**
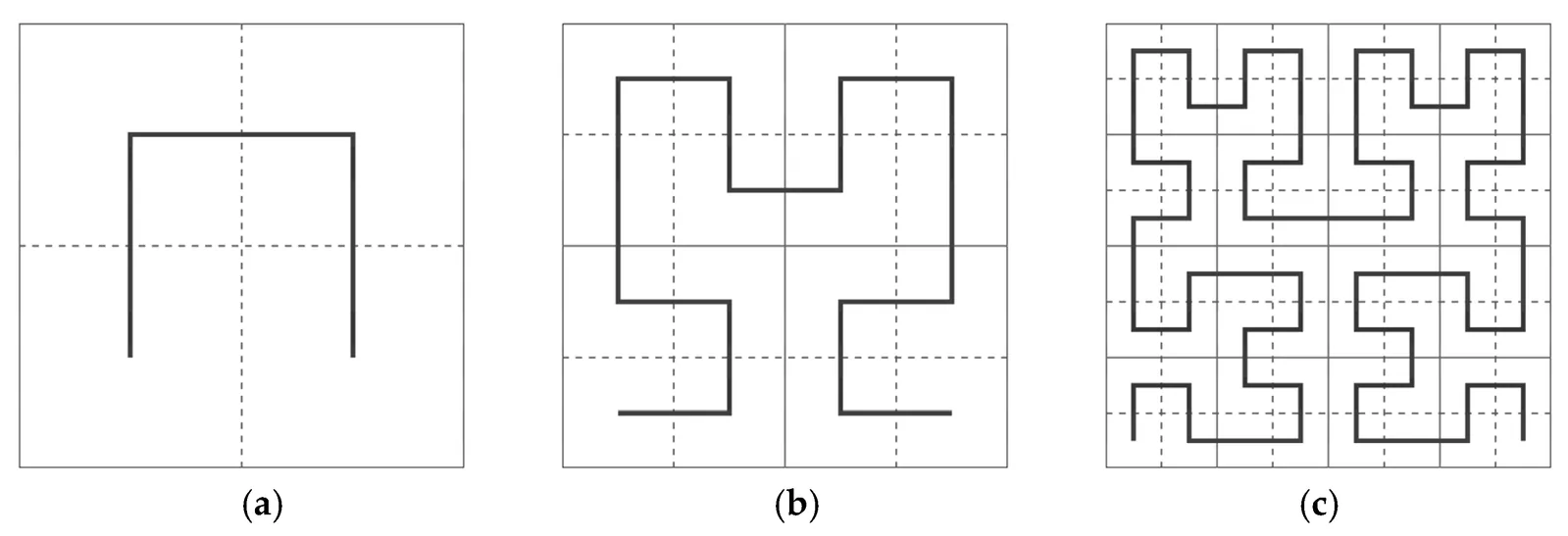
Hilbert fractal slot geometry: (**a**) first iteration, (**b**) second iteration, and (**c**) third iteration [18].

The Hilbert slot is used as the compact fractal radiator in this work because its space-filling geometry increases the effective current path within a limited physical area. As illustrated in Figure 1, the Hilbert geometry is generated through successive iterations, where the curve is folded inside a fixed square region. Increasing the iteration order enhances electrical compactness and supports multi-resonant/wideband behavior, which makes it suitable as a building block for the proposed four-port MIMO antenna [18].

**Step 1: Single-Element Optimization and Transition to the Four-Port HSMA**

**Figure 2:**
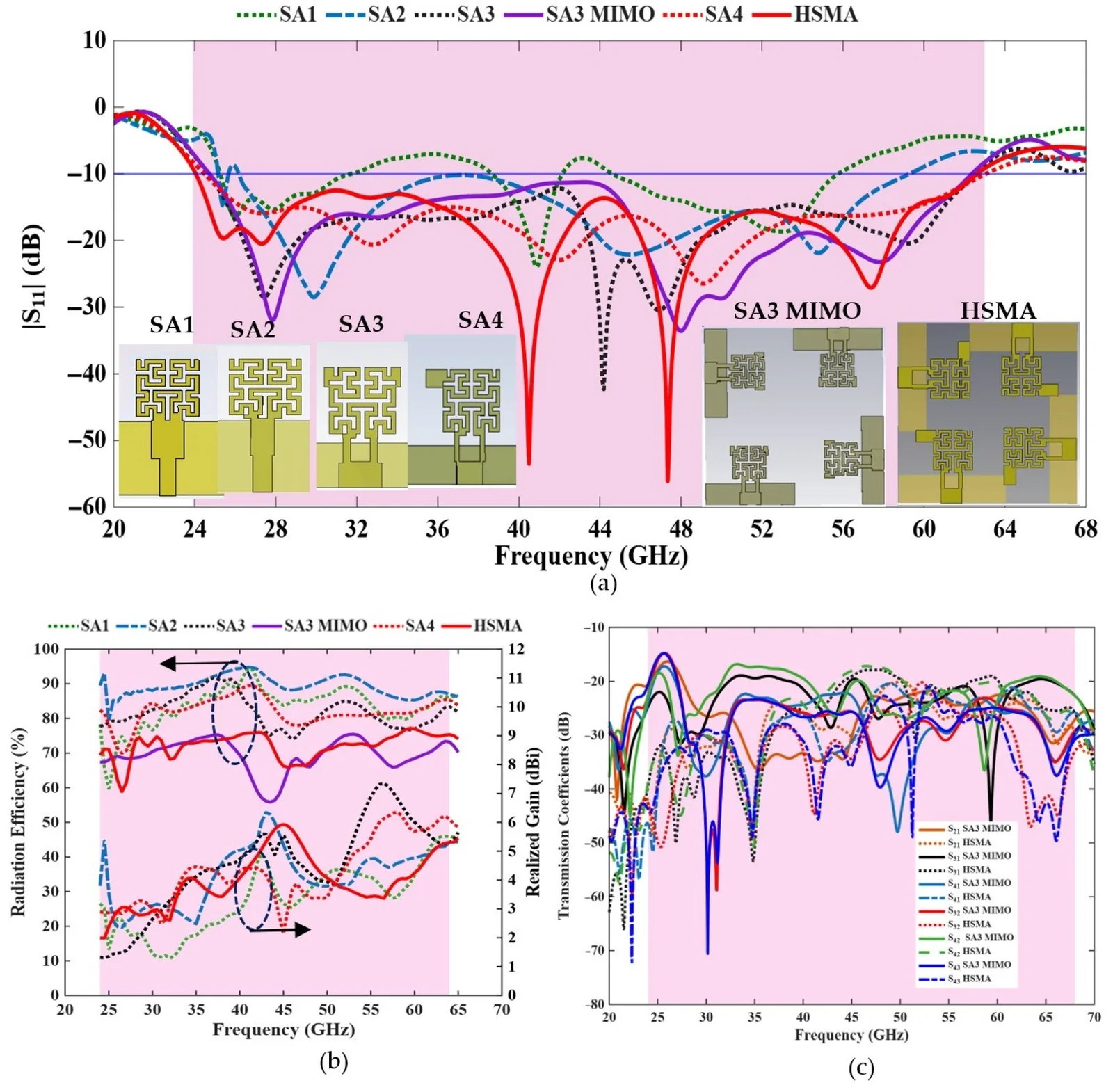
Design evolution and performance comparison of SA1–SA4, SA3-MIMO, and the proposed HSMA: (**a**) S_11_, (**b**) realized gain and radiation efficiency, and (**c**) transmission coefficients of SA3-MIMO and HSMA.

The design process starts with a compact third-order inverted Hilbert-slot element, denoted as SA1. As shown in Figure 2a, SA1 provides a wide impedance of S_11_ < −5.78 dB at 25.25–55.62 GHz, corresponding to a bandwidth of 30.37 GHz. However, the matching level is poor. Therefore, although SA1 confirms the potential of the Hilbert-slot path for wideband operation, further optimization is required to achieve practical impedance matching within the targeted mmWave range.

To improve the matching response, the stand position is modified, and an additional slot is introduced in the matching region, resulting in SA2. This modification improves the impedance matching to below −10 dB and extends the bandwidth to 32.98 GHz, covering 26.32–59.30 GHz. As shown in Figure 2b, the realized gain of SA2 also improves compared with SA1, increasing from 1.8 to 4.8 dBi for SA1 to 2.36–6.7 dBi for SA2. In addition, the radiation efficiency is enhanced from 61 to 95% for SA1 to 79–95% for SA2, confirming the effectiveness of the modified matching region.

Further improvement is achieved by extending both sides of the radiating patch, resulting in SA3. As shown in Figure 2a, this stage provides stronger impedance matching, with |S11| below −12.05 dB, and extends the operating range to 24.66–62.59 GHz, corresponding to a bandwidth of 37.93 GHz. As shown in Figure 2b, SA3 provides a realized gain of 1.0–7.3 dBi and a radiation efficiency of 73–93%. Although the minimum gain and efficiency are slightly reduced compared with SA2 at some frequencies, SA3 offers a wider operating bandwidth and stronger matching behavior, making it a suitable wideband single-element candidate.

Based on this improved response, a four-port SA3-MIMO configuration is first investigated by arranging four identical SA3 elements in a compact MIMO layout, as shown in the inset of Figure 2a. This intermediate stage is included to evaluate whether the SA3 element can preserve its wideband behavior after multi-port integration. The SA3-MIMO configuration provides impedance matching below −11.32 dB from 24.67 to 62.30 GHz, corresponding to a bandwidth of 37.63 GHz. However, although this result confirms the feasibility of SA3 for MIMO operation, the overall MIMO performance remains less favorable than that of the final proposed structure, particularly in terms of matching stability, realized gain, radiation efficiency, and inter-port isolation.

To improve the MIMO response, the SA3 element is further modified by replacing the two side extensions with one larger extension, resulting in SA4. This modification provides a more suitable element geometry for compact four-port integration. As shown in Figure 2a, the SA4 single element provides a wide operating range from 24.53 to 63.14 GHz, corresponding to a bandwidth of 38.61 GHz. Compared with SA3, SA4 slightly improves the bandwidth from 37.93 to 38.61 GHz and provides a more stable realized-gain response, varying from 2.63 to 5.90 dBi, compared with 1.00 to 7.30 dBi for SA3. However, this improvement is accompanied by a moderate reduction in radiation efficiency, from 73 to 93% for SA3 to 70–89.8% for SA4. This trade-off is acceptable because SA4 provides a more suitable geometry for compact four-port MIMO integration while maintaining wideband operation and reasonable radiation efficiency.

The final HSMA is then formed by arranging four identical SA4 elements in the proposed compact MIMO configuration. The proposed HSMA provides super-wideband operation from 24.05 to 62.64 GHz, corresponding to a bandwidth of 38.59 GHz, with impedance matching below −12.57 dB. Compared with the SA4 single element, the final HSMA preserves almost the same wide bandwidth while shifting the lower operating edge from 24.53 to 24.05 GHz, which is beneficial for covering the targeted mmWave operating band. More importantly, compared with the SA3-MIMO configuration, the proposed SA4-based HSMA improves the bandwidth from 37.63 to 38.59 GHz and provides better overall MIMO performance.

As shown in Figure 2b, the realized gain improves from 2.00 to 6.32 dBi for SA3-MIMO to 2.77–7.26 dBi for the proposed HSMA. Similarly, the radiation efficiency increases from 55 to 75% for SA3-MIMO to 58–75.7% for the proposed HSMA. In addition, the transmission-coefficient results in Figure 2c show that the proposed HSMA provides better inter-port isolation over the highlighted 24–68 GHz operating band. Therefore, the final SA4-based HSMA is selected as the proposed design because it provides the best compromise between wideband impedance matching, compact MIMO integration, realized gain, radiation efficiency, and inter-port isolation.

**Step 2: Analysis of the Optimized SA4 Single Element**

**Figure 3:**
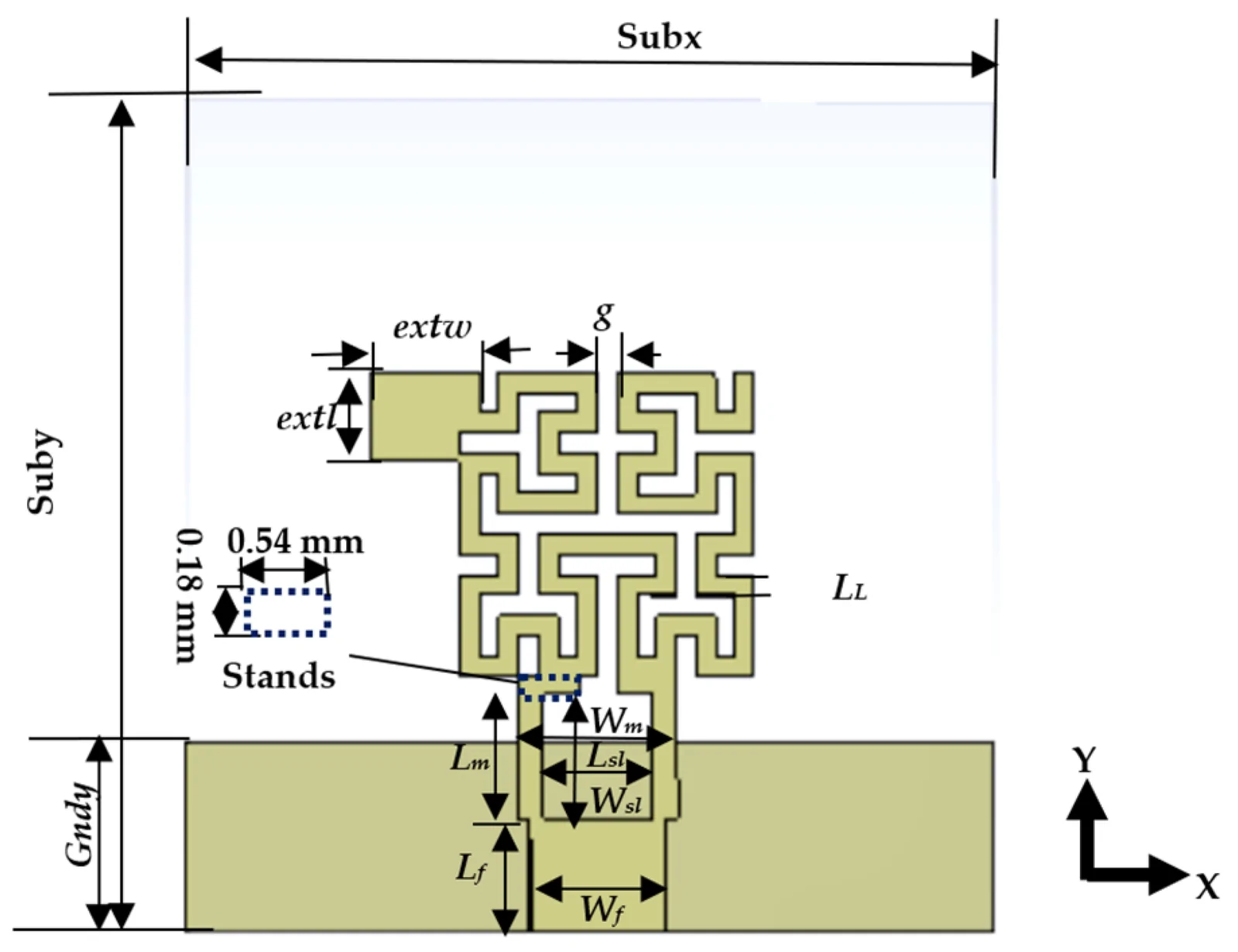
Layout of the optimized SA4 single element with key geometrical parameters.

After selecting SA4 as the final single-element radiator, a dedicated analysis is carried out to further clarify its electromagnetic behavior before integration into the four-port HSMA. This analysis is included to address the role of the single element in achieving the wideband response of the proposed antenna. The SA4 element is investigated in terms of reflection coefficient, realized gain, radiation efficiency, surface-current distribution, 3D radiation patterns and key geometrical parameters. Since SA4 represents the actual element used to construct the final HSMA, this analysis provides a direct explanation of how the modified Hilbert-slot path and the single large extension contribute to impedance matching, current distribution, and radiation performance over the highlighted operating band. The SA4 layout is shown in Figure 3. It is designed on a Rogers RT/Duroid 5880 substrate with an overall size of 7.31 × 7.31 mm^2^. The main geometrical parameters of the SA4 element are also indicated in Figure 3, including the Hilbert line length (*L_L_* = g), feedline length (*L_f_*), feedline width (*W_f_*), matching-line length (*L_m_*), matching-line width (*W_m_*), slot length (*L_sl_*), slot width (*W_sl_*), extension length (*extl*), extension width (*extw*), substrate dimensions (*Subx* and *Suby*), partial ground length (*Gndy*), and gap between the Hilbert segments. Introducing these parameters at the single-element stage allows the optimization process to be directly linked to the physical structure used in the final HSMA.

**Figure 4:**
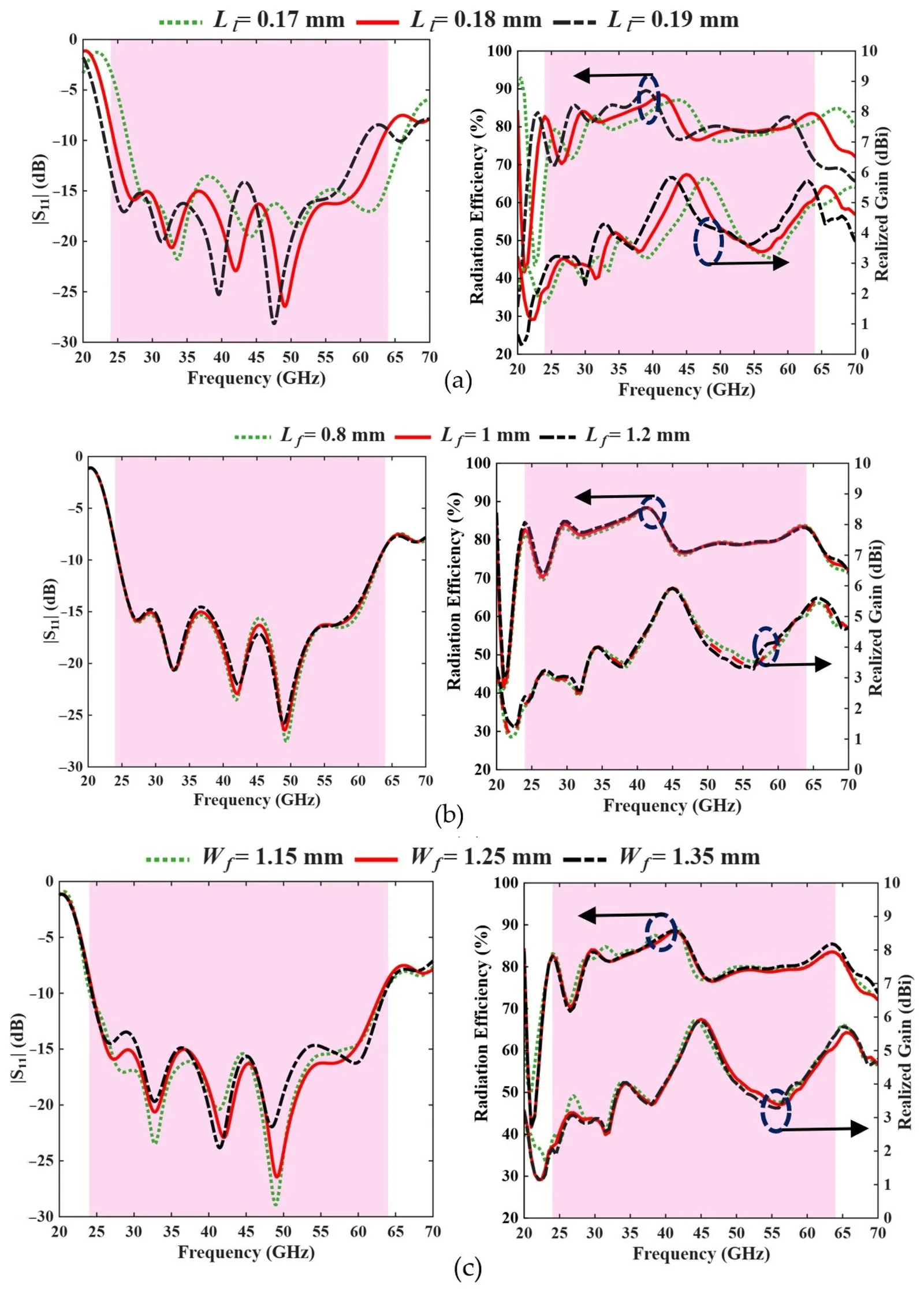

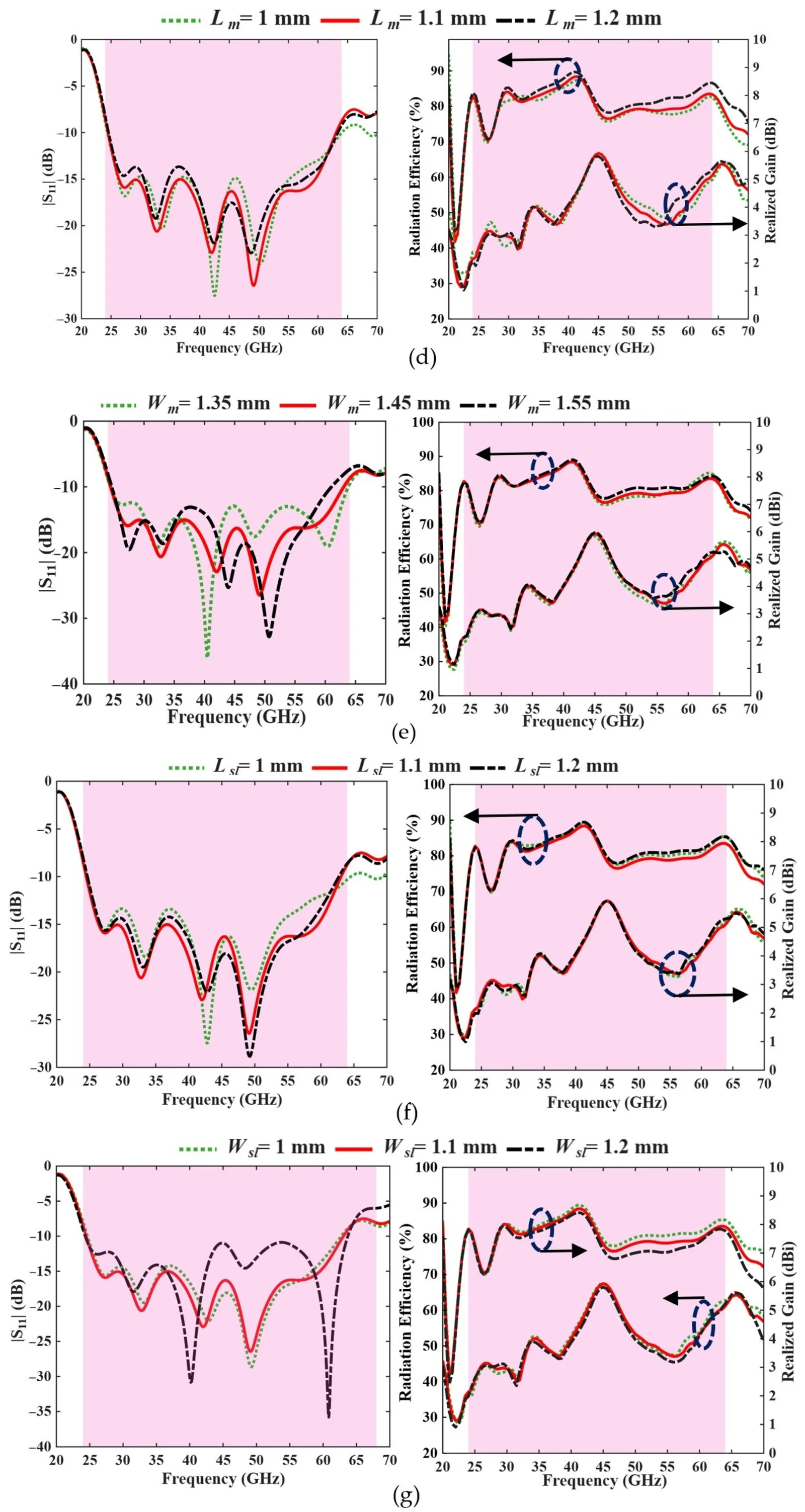

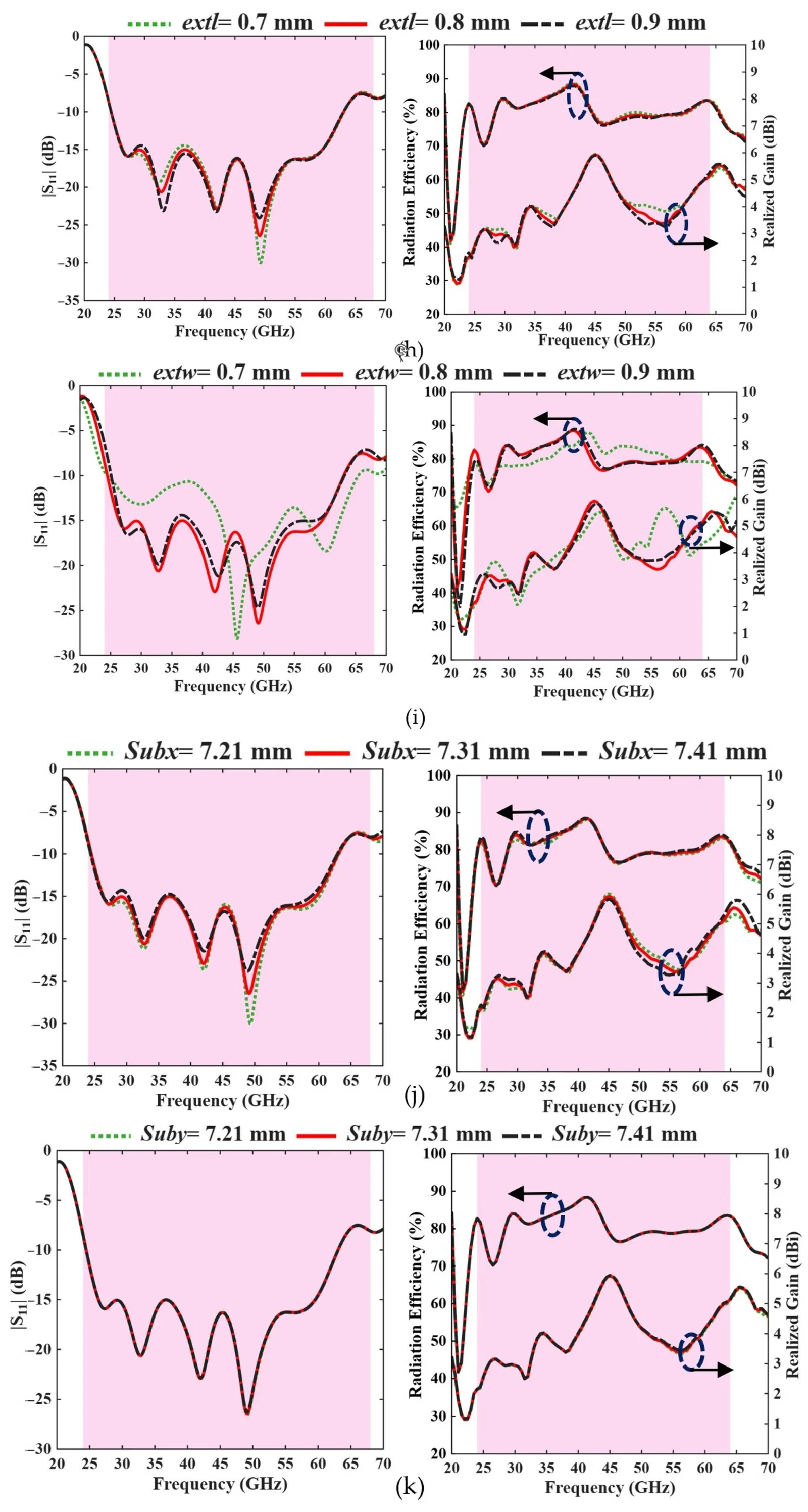

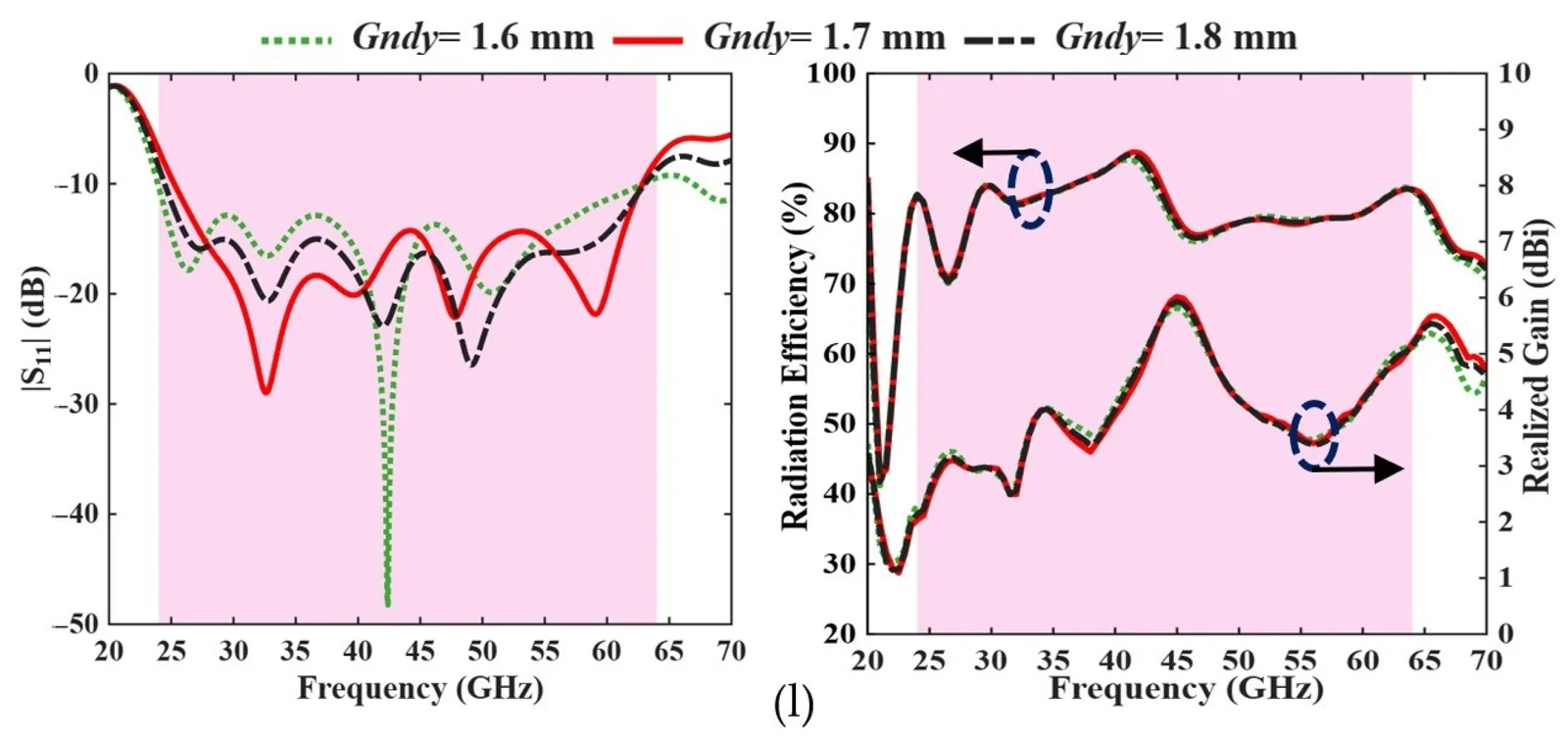
Parametric study of SA4 single-element simulated S_11_, gain, and radiation efficiency responses for variations in: (**a**) *L_L_*, (**b**) *L_f_*, (**c**) *W_f_*, (**d**) *L_m_*, (**e**) *W_m_*, (**f**) *L_sl_*, (**g**) *W_sl_*, (**h**) *extl*, (**i**) *extw*, (**j**) *Subx*, (**k**) *Suby* and (**l**) *Gndy*.

Figure 4 presents the parametric study of the optimized SA4 single element. The study is carried out to clarify how the main geometrical parameters affect the impedance response, realized gain, and radiation efficiency before the element is integrated into the four-port HSMA. The pink shaded region indicates the targeted operating band from 24 to 68 GHz.

The Hilbert line length, *L_L_* (Figure 4a), has a clear influence on the resonance positions and impedance matching across the operating band. Increasing *L_L_* shifts the resonant behavior and modifies the strength of the matching minima, confirming that the Hilbert-slot path controls the effective current length of the radiator. Among the investigated values, *L_L_* = 0.18 mm provides the best overall compromise between wideband matching, stable realized gain, and acceptable radiation efficiency. The corresponding efficiency variation is mainly attributed to the change in current confinement along the Hilbert-slot path; when the current path becomes better matched, more input power is radiated, while excessive current concentration around narrow slot sections can increase loss at some frequencies.

The feedline length, *L_f_* (Figure 4b), has a smaller effect on the reflection coefficient compared with *L_L_*, indicating that the selected feedline length mainly fine-tunes the input transition rather than changing the overall resonance mechanism. Although *L_f_* = 0.9 mm and *L_f_* = 1.1 mm provide similar wideband behavior, *L_f_* = 1 mm is selected because it maintains stable |S_11_|, realized gain, and radiation efficiency while preserving compactness. The efficiency remains relatively stable for the investigated *L_f_* values because this parameter mainly affects the feeding transition rather than the main radiating slot path.

The feedline width, *W_f_* (Figure 4c), affects the impedance transition between the feed and the Hilbert-slot radiator. Its variation changes the matching level, especially around the mid-band and upper-band regions. The selected value of *W_f_* = 1.25 mm provides a balanced response, with wide impedance matching and stable radiation performance over the highlighted operating band. The observed efficiency fluctuation occurs because changing *W_f_* modifies the input coupling and local current density at the feed junction, which can either improve power transfer to the radiator or increase localized loss.

The matching-line length, *L_m_* (Figure 4d), and matching-line width, *W_m_* (Figure 4e), show stronger effects on the impedance response because these parameters directly control the coupling and transition between the feed region and the radiating Hilbert-slot path. The results show that inappropriate values can improve matching at one resonant region while degrading another part of the band. Therefore, *L_m_* = 1.1 mm and *W_m_* = 1.45 mm are selected to provide a balanced wideband response rather than a narrow deep resonance at a single frequency. Their effect on efficiency follows the same behavior: values that produce smoother current transfer from the feed to the slot generally maintain higher efficiency, while over-coupled or poorly matched cases show reduced efficiency at some frequencies.

The slot length, *L_sl_* (Figure 4f), and slot width, *W_sl_* (Figure 4g), also play an important role in controlling the current path and the location of the resonant modes. Increasing these parameters changes the effective slot loading and shifts the impedance minima across the band. The selected values provide a good compromise between impedance matching, realized gain, and radiation efficiency, confirming that the slot dimensions help support the super-wideband behavior of the SA4 element. The efficiency variations are expected because the slot dimensions affect both the radiating path and the strength of surface-current concentration along the slot edges.

The extension length, *extl* (Figure 4h), mainly fine-tunes the upper-band response and contributes to the wideband behavior of the modified SA4 structure. Although the investigated values show similar radiation efficiency and realized-gain trends, *extl* = 0.8 mm gives the most balanced impedance response across the operating band. This confirms that the single large extension is not arbitrary; rather, it is introduced to improve the impedance response and make the element more suitable for compact MIMO integration. The slight efficiency changes associated with *extl* are due to the redistribution of current over the extension region, which affects the balance between radiated power and localized conductive/dielectric losses at higher frequencies. The extension width, *extw* (Figure 4i), also has a noticeable effect on the SA4 response. Changing *extw* modifies the effective width of the extension region and therefore alters the current distribution and impedance behavior across the operating band. The selected value of *extw* provides the most balanced response in terms of wideband impedance matching, realized gain, and radiation efficiency.

The substrate dimensions, *Subx* (Figure 4j) and Suby (Figure 4k), influence the electromagnetic boundary around the compact SA4 radiator. Changing *Subx* mainly affects the lateral fringing fields and edge interaction of the substrate. Since the main Hilbert-slot path and feed region remain unchanged, the effect of *Subx* on ∣S_11_∣ is relatively limited compared with the slot and matching-line parameters. However, small changes in the resonance depths can still be observed because the substrate width modifies the surrounding dielectric loading and current spreading near the antenna edges. The realized gain and radiation efficiency remain generally stable for the investigated *Subx* values, indicating that *Subx* mainly provides structural compactness while having only a secondary effect on the radiation behavior. The parameter *Suby* has a more noticeable effect on the impedance response because it controls the substrate extension in the direction of the feed and partial ground plane. Varying *Suby* changes the effective dielectric loading, the current return path, and the field distribution around the lower part of the radiator. As a result, slight shifts in the ∣S_11_∣ minima and matching levels can occur across the operating band. The selected *Suby* value provides the best compromise between wideband impedance matching, stable realized gain, and acceptable radiation efficiency. The gain and efficiency variations remain moderate, showing that *Suby* mainly contributes to fine-tuning the antenna matching rather than significantly changing the main radiation mechanism.

The partial ground length, *Gndy* (Figure 4l), plays an important role in controlling the feed-to-radiator coupling and the impedance transition of the SA4 element. Changing *Gndy* modifies the ground-plane current distribution and the capacitive coupling between the feed region and the Hilbert-slot radiator. Therefore, this parameter has a clear influence on the ∣S_11_∣ response, especially in the mid- and upper-frequency regions. An unsuitable *Gndy* value can improve matching at one frequency range while degrading another part of the band. The optimized *Gndy* value is selected because it maintains wideband matching while preserving stable realized gain and radiation efficiency. The observed efficiency changes are mainly associated with the redistribution of current on the partial ground plane and the resulting balance between radiated power and local losses.

Overall, the parametric study shows that the optimized SA4 dimensions are selected based on a balanced response rather than the deepest reflection-coefficient minimum. The final dimensions provide wide impedance matching, stable realized gain, and acceptable radiation efficiency across the targeted band. The observed efficiency fluctuations are mainly related to changes in current concentration, feed-to-slot coupling, and loss distribution at mmWave frequencies, which will be further explained through the surface-current distributions in the following section.

**Figure 5:**
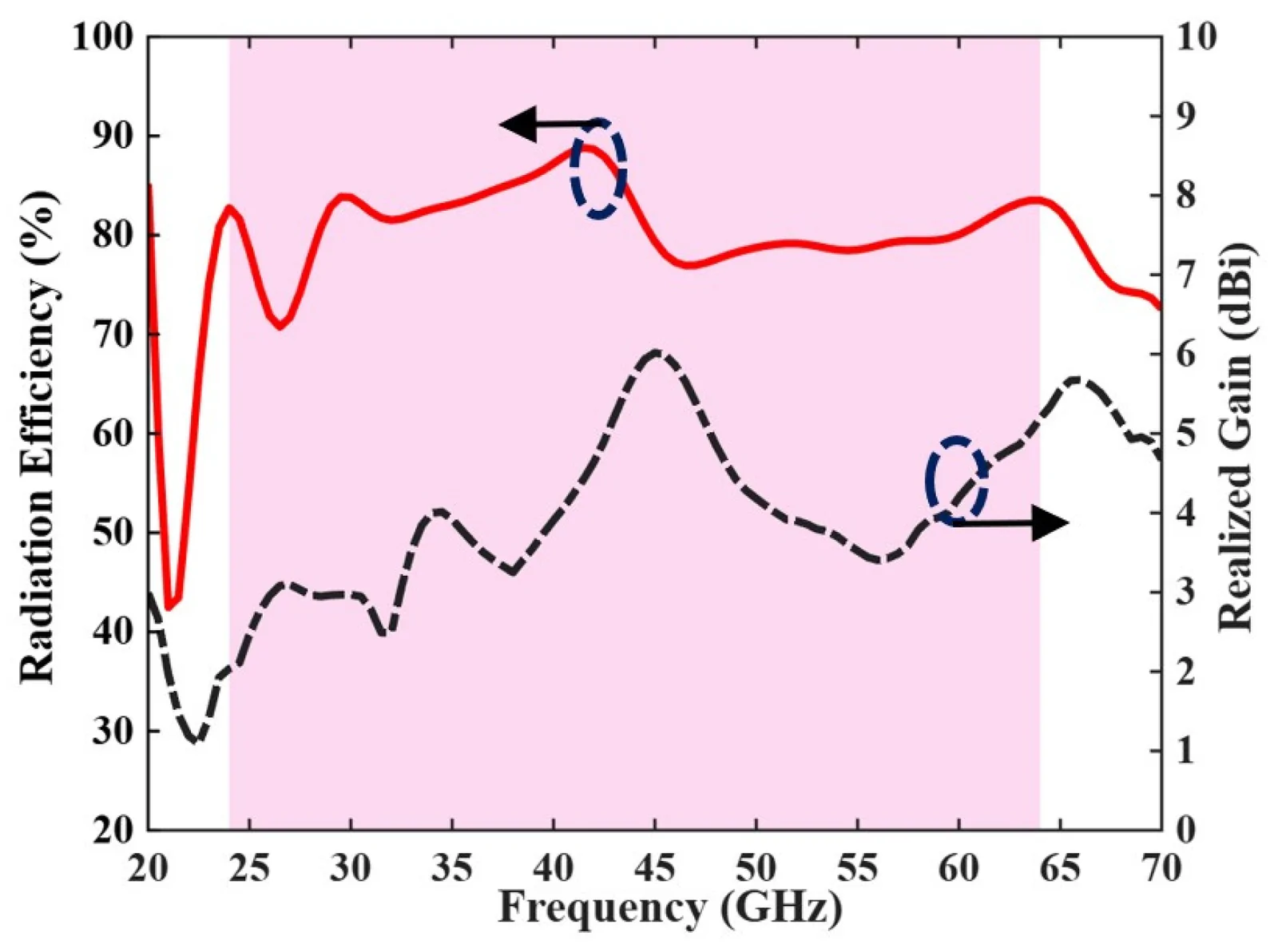
Gain and radiation efficiency responses for the optimized SA4.

The optimized dimensions in Figure 4 are selected to provide a balanced response in terms of impedance matching, realized gain, and radiation efficiency. To better examine the radiation-performance behavior of the final single element, Figure 5 presents the realized gain and radiation efficiency of the optimized SA4 element over the operating band. This figure is included before the surface-current analysis to show the frequency regions where the gain and efficiency vary, and to support the physical interpretation of these variations.

**Figure 6:**
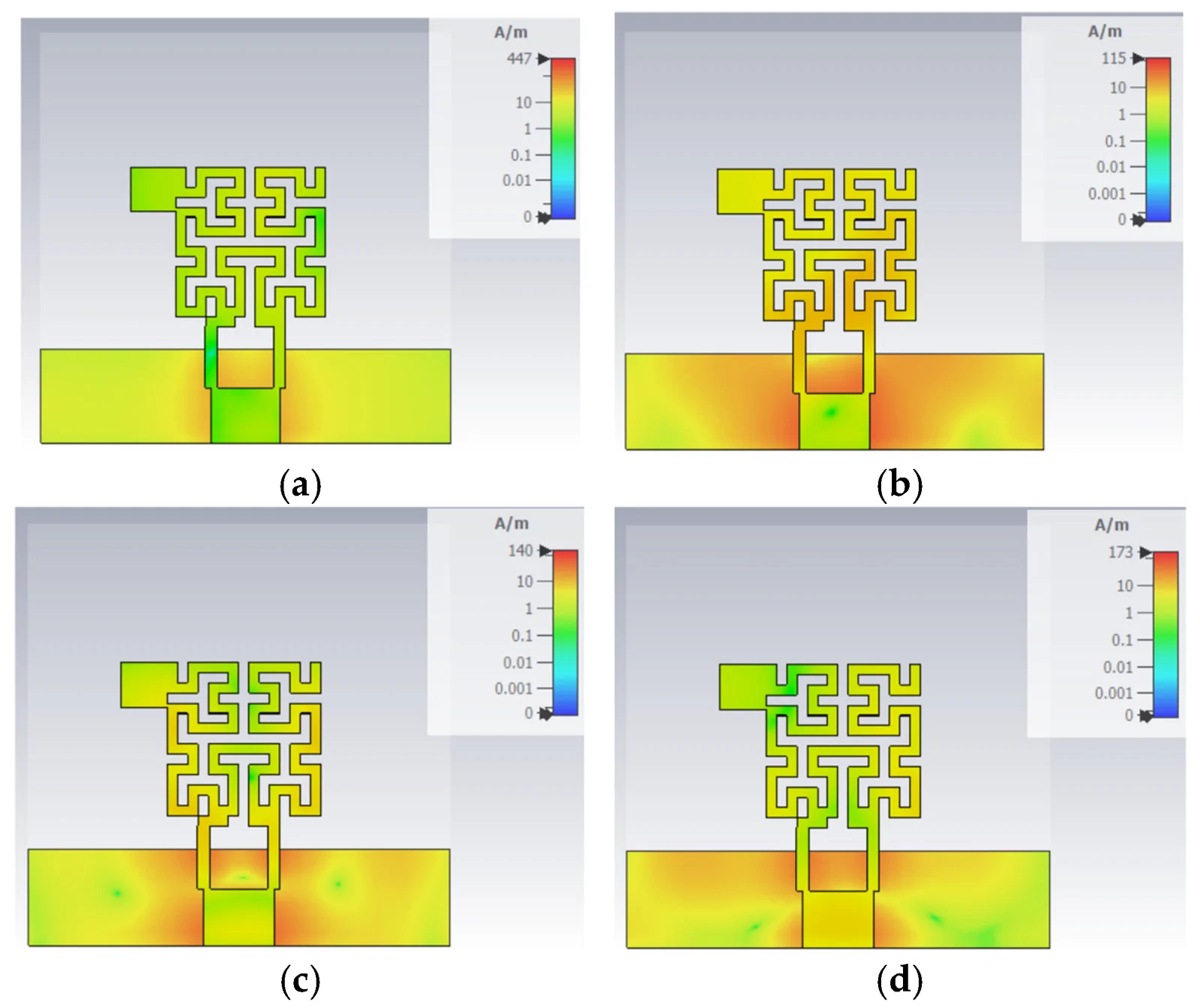
Surface current for the optimized SA4 single element at: (**a**) 28 GHz, (**b**) 40 GHz, (**c**) 50 GHz and (**d**) 60 GHz.

To explain the behavior observed in Figure 5, the surface-current density of the optimized SA4 single element is analyzed in Figure 6 at 28, 40, 50, and 60 GHz. These frequencies are selected to represent the lower, middle, upper-middle, and upper regions of the highlighted 24–68 GHz operating band. They are not chosen only as isolated resonance points, but rather to examine how the current distribution changes across the band and how these changes relate to the realized-gain and radiation-efficiency responses. This analysis helps clarify the role of the feedline, Hilbert-slot path, slot edges, and large extension in supporting the wideband operation of the SA4 single element.

Figure 5 presents the realized gain and radiation efficiency of the optimized SA4 single element. The results show that the SA4 element maintains useful radiation performance across the highlighted operating band. The radiation efficiency remains relatively high over most of the band, while the realized gain varies with frequency because of the frequency-dependent current distribution, impedance matching, and radiation-mode formation. The gain increases in the middle and upper parts of the band, whereas local reductions are observed at some frequencies due to changes in current confinement and radiation efficiency. Therefore, the gain and efficiency trends in Figure 5 are further interpreted using the surface-current distributions shown in Figure 6.

Figure 6 shows the surface-current density of the optimized SA4 single element at 28, 40, 50, and 60 GHz. At 28 GHz, the current is mainly distributed around the feed transition, lower slot region, and central Hilbert-slot path, confirming that the lower-band operation is strongly supported by the feed-to-slot coupling and the effective current length of the Hilbert geometry. At 40 GHz, the current becomes more distributed along the Hilbert-slot edges and the large extension, indicating that the modified slot route contributes more strongly to the mid-band response. At 50 GHz, the current is concentrated around the feed region, inner slot path, and lower part of the extension, which explains the continued wideband matching and the corresponding gain variation observed in Figure 5. At 60 GHz, the current spreads over both the slot path and the extension region, confirming that the upper-band operation is supported by the combined contribution of the Hilbert-slot radiator and the added large extension.

Overall, the surface-current distributions confirm that the wideband response of SA4 is not only produced by the feedline. Instead, it results from the combined effect of the feed transition, Hilbert-slot path, slot edges, and large extension. The changes in current concentration across the selected frequencies also explain the observed variations in realized gain and radiation efficiency. Frequencies with smoother current transfer from the feed to the radiating slot generally maintain better radiation performance, while stronger local current concentration around narrow slot edges or transition regions can increase loss and produce local reductions in efficiency.

**Figure 7:**
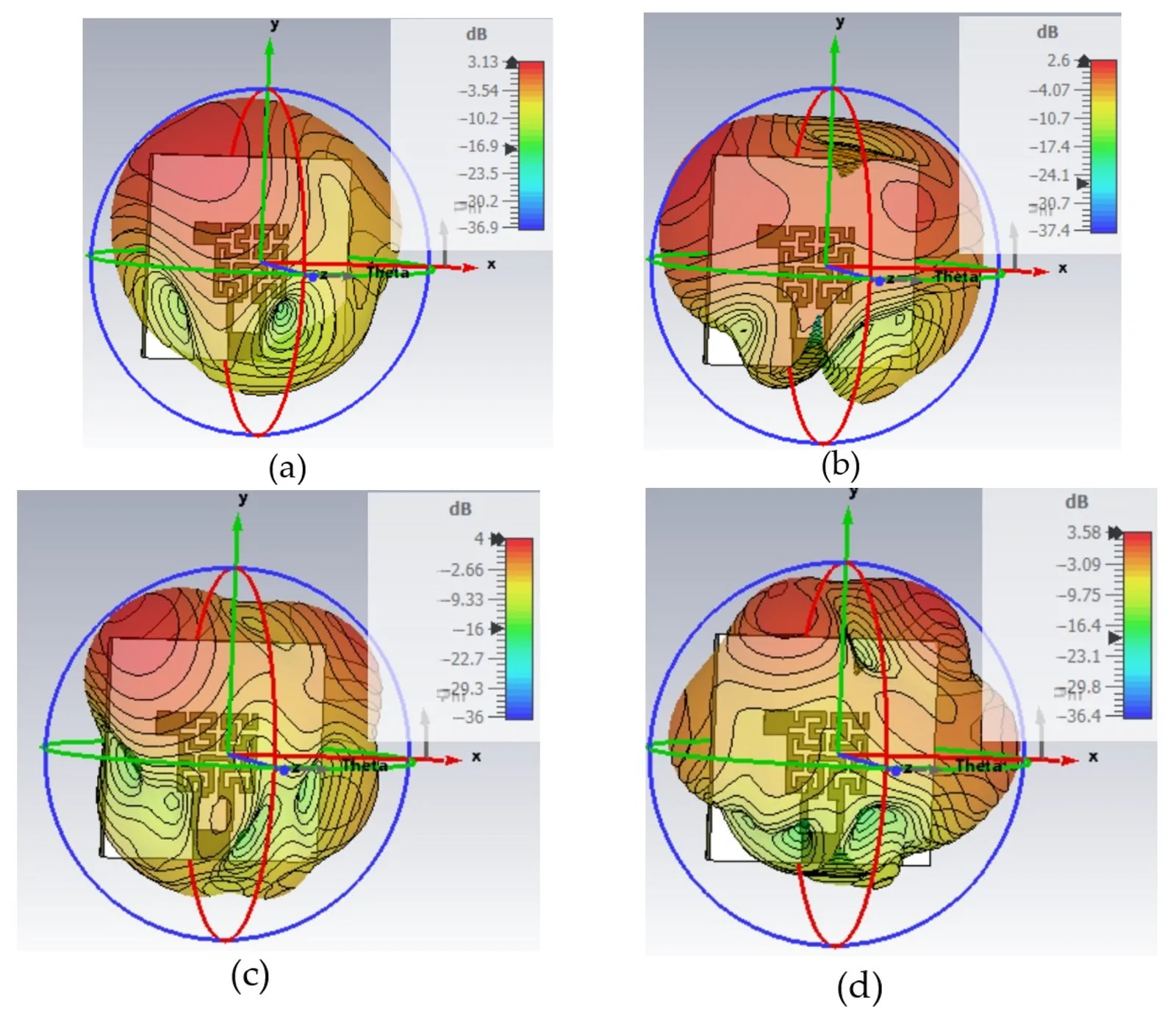
3D radiation patterns for the optimized SA4 single element at: (**a**) 28 GHz, (**b**) 40 GHz, (**c**) 50 GHz and (**d**) 60 GHz.

After examining the surface-current behavior, the corresponding 3D realized-gain patterns are presented in Figure 7 at the same selected frequencies. Using the same frequencies allows a direct correlation between the current distribution and the resulting radiation behavior. The 3D patterns are presented in terms of realized gain rather than directivity because realized gain includes both radiation losses and input-mismatch effects, thereby providing a more practical representation of the antenna performance.

At 28 GHz, the radiation is mainly supported by the feed-to-slot coupling and the central Hilbert-slot path, resulting in stable lower-band radiation behavior. At 40 GHz, the current becomes more distributed along the Hilbert-slot edges and extension region, which contributes to the increase in realized gain observed in Figure 5. The radiation stays consistent at 50 GHz due to the even distribution of the current in the feed region, slot path, and lower extension section, which explains the wide band range. At 60 GHz, the current distributes over a larger section of the slot and extension zones, allowing for better radiation performance in the top half of the operating range.

The 3D patterns also support the gain and radiation-efficiency trends observed in Figure 5. Frequencies exhibiting smoother and more distributed current flow generally correspond to improved realized gain and stable radiation efficiency, whereas stronger local current concentration is associated with the slight gain and efficiency reductions observed at some frequencies. Although some variation in the radiation shape is observed across the operating band, the patterns confirm that the optimized SA4 element maintains stable wideband radiation behavior. Therefore, the combined gain, radiation-efficiency, surface-current, and 3D-pattern analyses confirm that the wideband response of the SA4 element is supported by the coordinated contribution of the feed transition, Hilbert-slot path, slot edges, and large extension before its integration into the four-port HSMA.

**Step 3: Four-Port HSMA Configuration**

**Figure 8:**
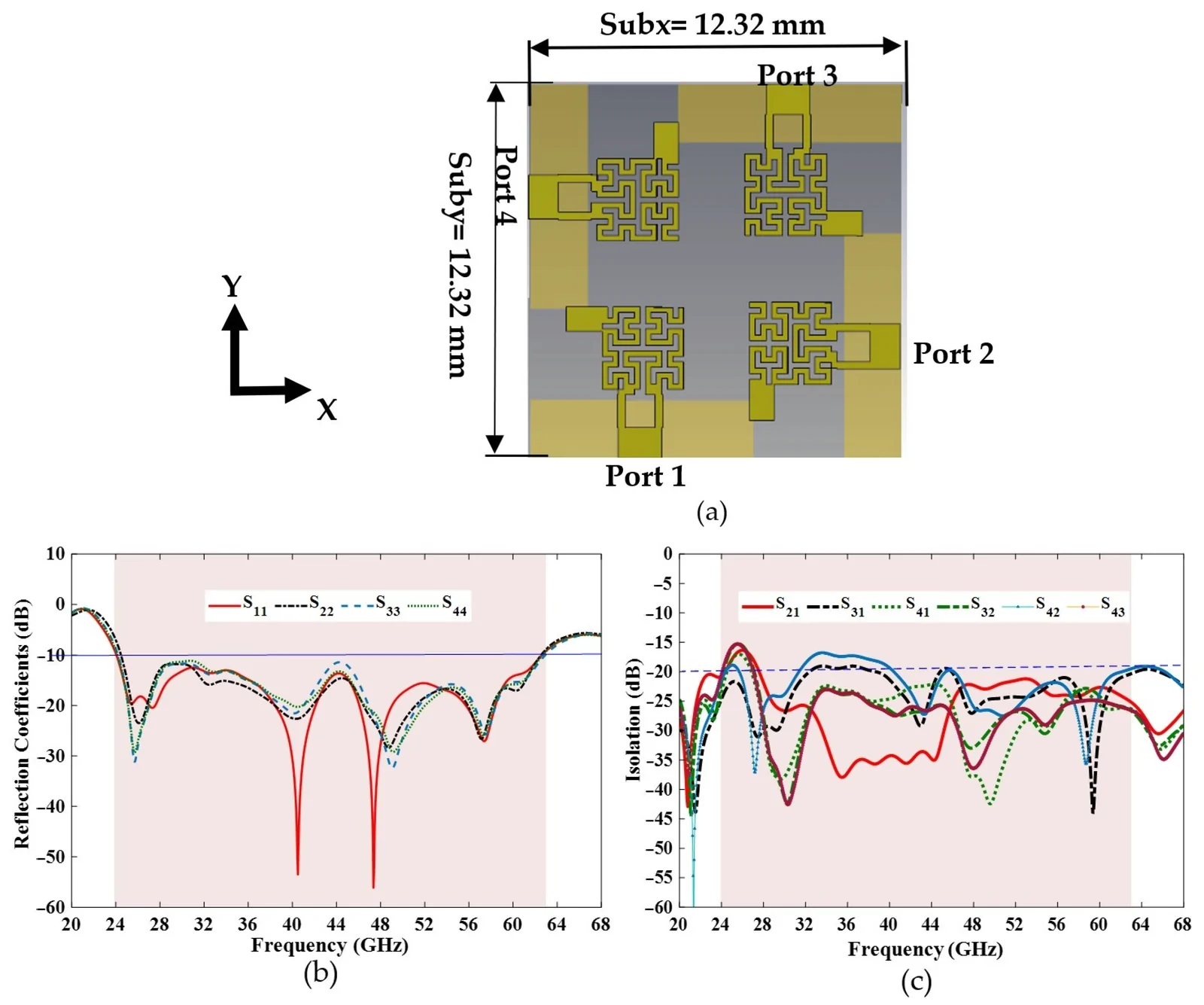
Proposed four-port Hilbert-slot MIMO antenna: (**a**) final HSMA layout, (**b**) simulated reflection coefficients, (**c**) simulated inter-port isolation.

After completing the single-element analysis of SA4, four identical SA4 elements are arranged to form the proposed four-port HSMA, as shown in Figure 8a. The final MIMO structure is also designed on a Rogers RT/Duroid 5880 substrate with an overall size of 12.32 × 12.32 mm^2^. The four elements are placed in a compact symmetrical arrangement to preserve the super-wideband impedance response while reducing mutual coupling between adjacent ports.

The simulated reflection coefficients of the four ports are shown in Figure 8b. The proposed HSMA maintains super-wideband impedance matching from 24.05 to 62.64 GHz, corresponding to a bandwidth of 38.57 GHz. This confirms that the transition from the optimized single element to the four-port MIMO configuration does not degrade the wideband behavior. The isolation performance is shown in Figure 8c, where the transmission coefficients between the ports remain at acceptable low levels across the operating band. This indicates that the proposed compact arrangement provides suitable MIMO operation for wearable mmWave telemetry applications.

**Step 4: Parametric Analysis and Performance Enhancement of HSMA**

**Figure 9:**
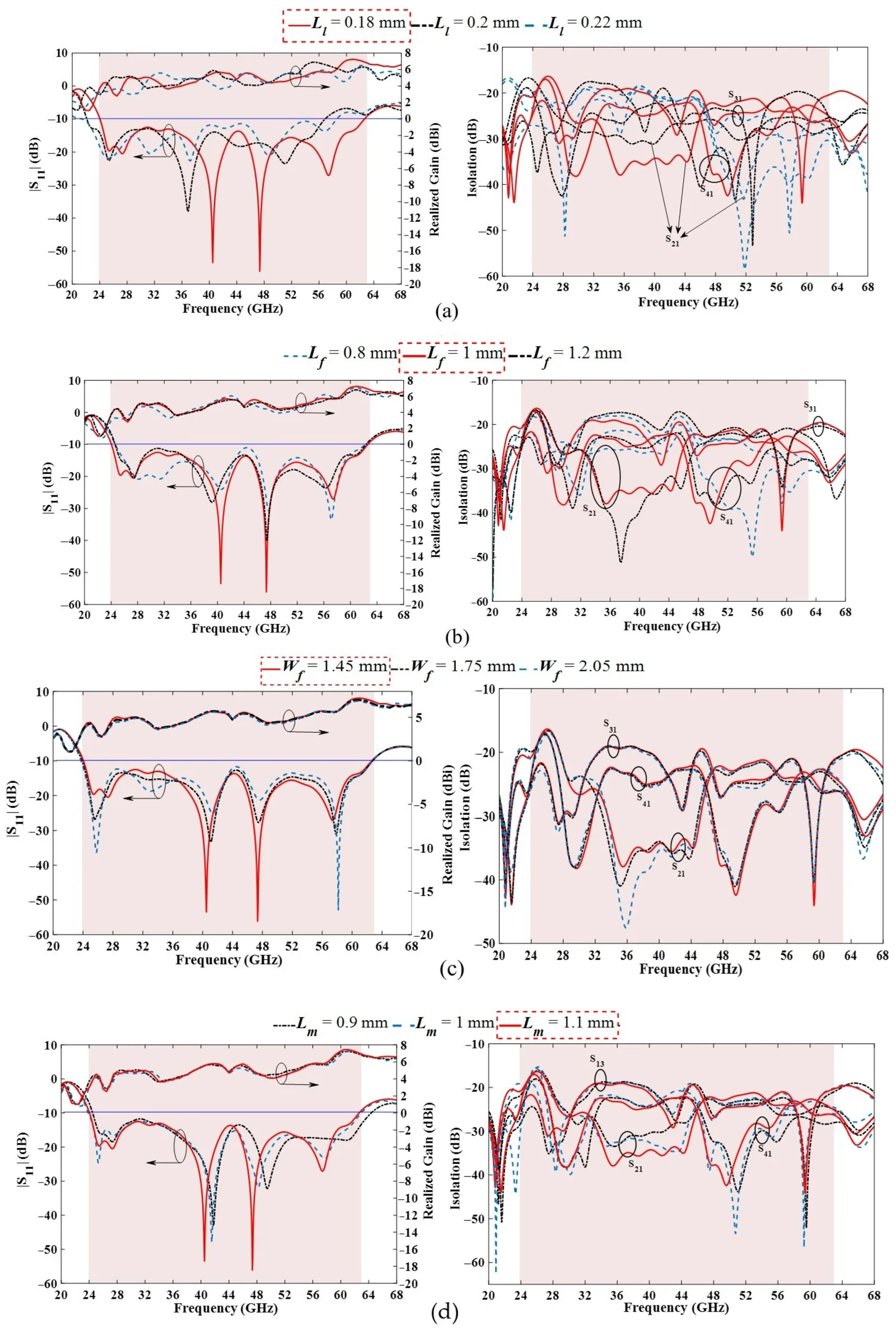

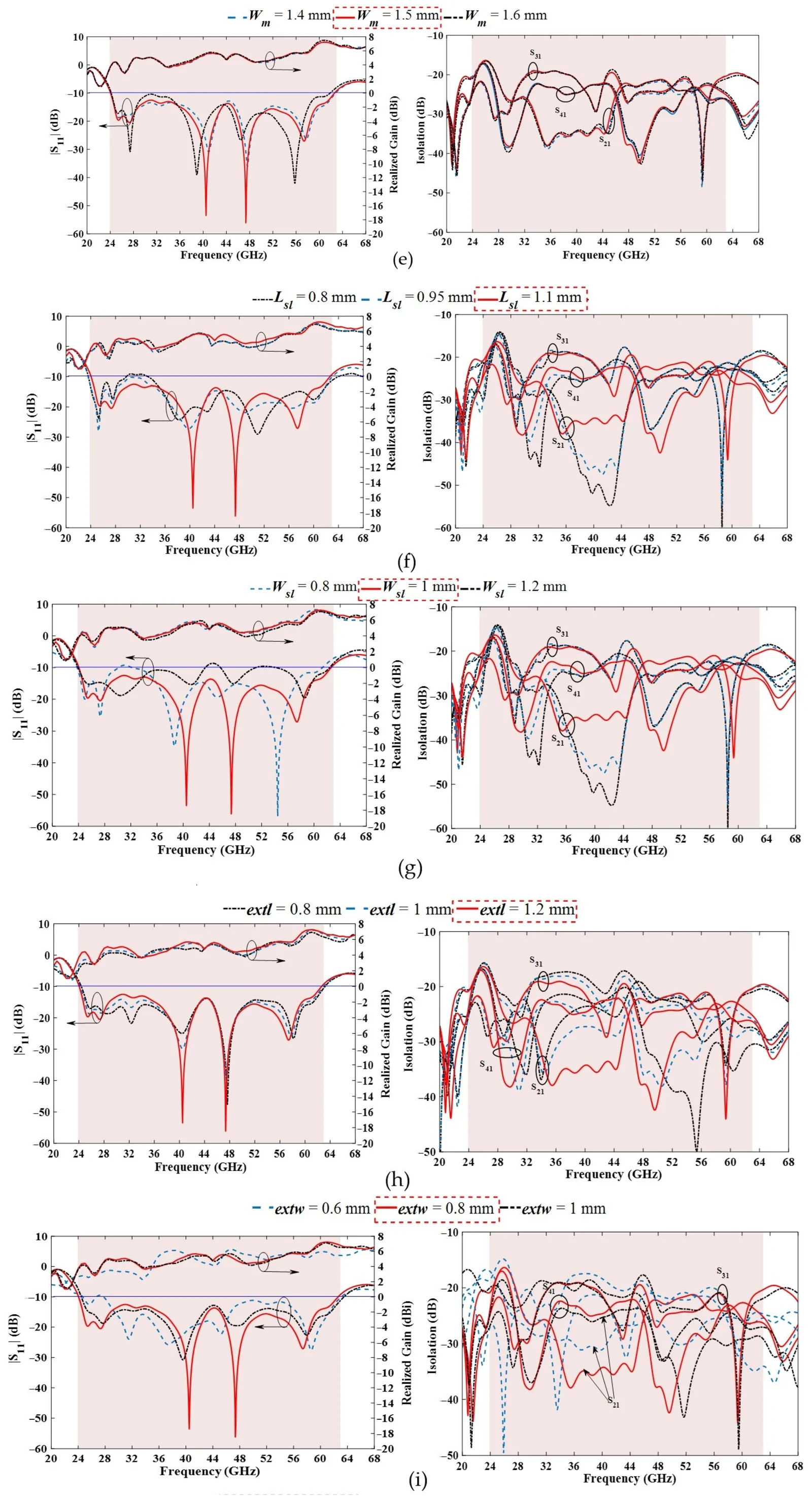

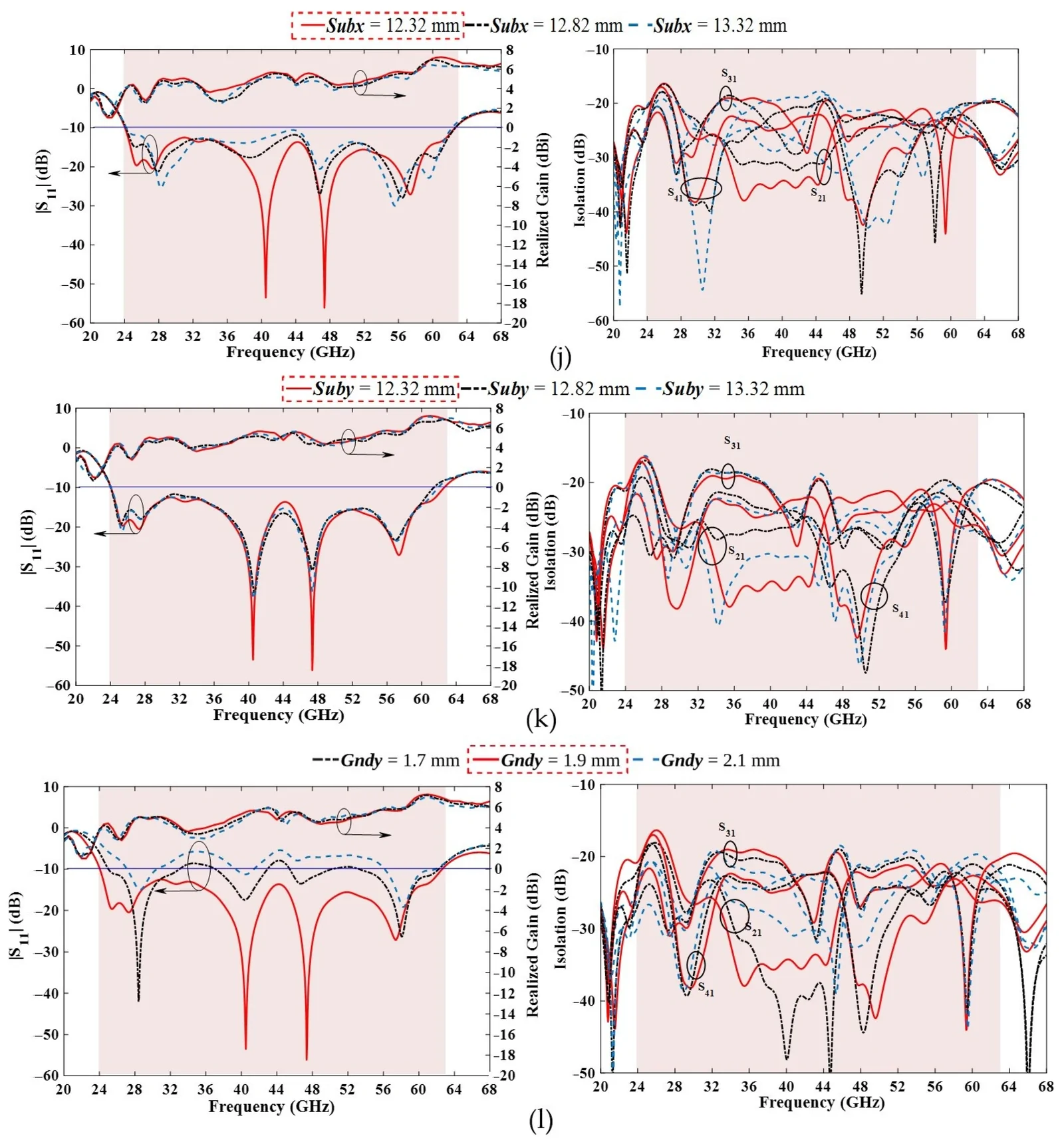
Parametric study of the proposed four-port HSMA showing simulated S_11_, gain, and isolation responses for variations in: (**a**) *L_L_*, (**b**) *L_f_*, (**c**) *W_f_*, (**d**) *L_m_*, (**e**) *W_m_*, (**f**) *L_sl_*, (**g**) *W_sl_*, (**h**) *extl*, (**i**) *extw*, (**j**) *Subx*, (**k**) *Suby* and (**l**) *Gndy*.

After the proposed four-port HSMA is developed, the same geometrical parameters introduced for the SA4 single element are re-evaluated in the MIMO configuration. This parametric study shown in Figure 9 investigates their influence on the final HSMA performance, including impedance matching, bandwidth, realized gain, radiation efficiency, compactness, and inter-port isolation.

The parametric analysis shows that the Hilbert line length has a strong influence on the operating band because it directly controls the effective current path of the fractal slot. It is shown that a small variation in *L_L_* produces a significant frequency shift, confirming that this parameter is crucial in order to maintain the required FR2 super-wideband response. The optimized value of *L_L_* = 0.18 mm provides the best compromise between the matching, bandwidth and gain, achieving operation from 24.66 to 62.59 GHz with a bandwidth of 37.93 GHz and a maximum gain of 7.35 dBi.

The feeding and matching parameters also play an important role in stabilizing the impedance response of the antenna. The selected length of the feedline *L_f_* = 1 mm provides good matching with the compactness preserved. A longer feedline could improve the gain or matching at some frequencies, but the selected value avoids unnecessary size increase. Similarly, *W_f_* = 1.75 mm is selected since it gives stable matching in the desired band. In the matching section, *L_m_* = 1 mm and *W_m_* = 1.4 mm provide a proper compromise between bandwidth, compactness, and impedance stability.

The slot dimensions also contribute to the wideband response. The improved matching and gain over the operating band are achieved by the optimized values of *L_sl_* = 0.95 mm and *W_sl_* = 0.8 mm. The extension parameters also play a role in the final impedance response. The side extensions improve the current distribution and enhance the antenna performance by choosing *extl* = 0.2 and *extw* = 0.8 mm as the optimum values. Another important parameter is the partial ground length that affects the impedance bandwidth and the coupling behavior. The selected value of *Gndy* = 2 mm provides better matching across the FR2 band compared with the other investigated values.

The substrate dimensions are selected to preserve compactness while maintaining the desired wideband performance. The optimized values of *Subx* = *Suby* = 7.31 mm are used for the single radiating element, and the final four-port HSMA is then arranged within an overall footprint of 12.32 × 12.32 mm^2^. Based on the optimized single element, the proposed four-port HSMA achieves super-wideband operation from 24.05 to 62.64 GHz, corresponding to a bandwidth of 38.57 GHz. This shows that the transition from the optimized single element to the four-port MIMO configuration preserves the wideband response while enabling MIMO functionality.

The effects of the main geometrical parameters on the reflection coefficient, gain, and inter-port isolation are summarized in Table 1, while the corresponding simulated responses are shown in Figure 9. The selected dimensions provide the best compromise between super-wideband impedance matching, stable gain behavior, low mutual coupling, and compact MIMO layout.

**Figure 10:**
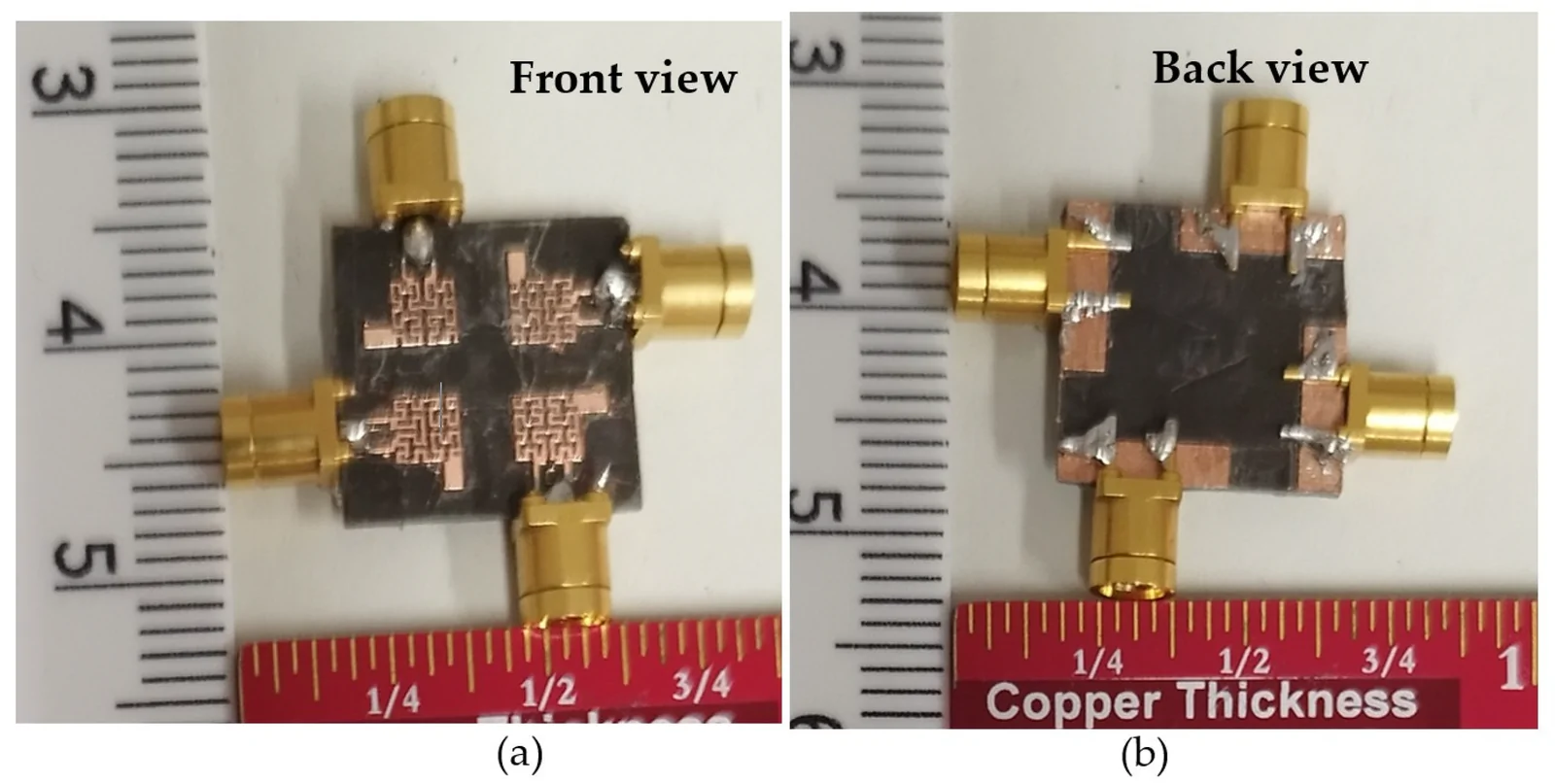
Fabricated prototype of the proposed four-port HSMA: (**a**) front view and (**b**) back view.

The fabricated prototype of the proposed four-port HSMA, realized using the optimized dimensions obtained from the parametric study, is shown in Figure 10. The front view illustrates the four Hilbert-slot radiating elements and feeding ports, while the back view shows the corresponding ground/metallization layout.

**Step 5: Surface Current Distribution and MIMO Coupling Mechanism**

**Figure 11:**
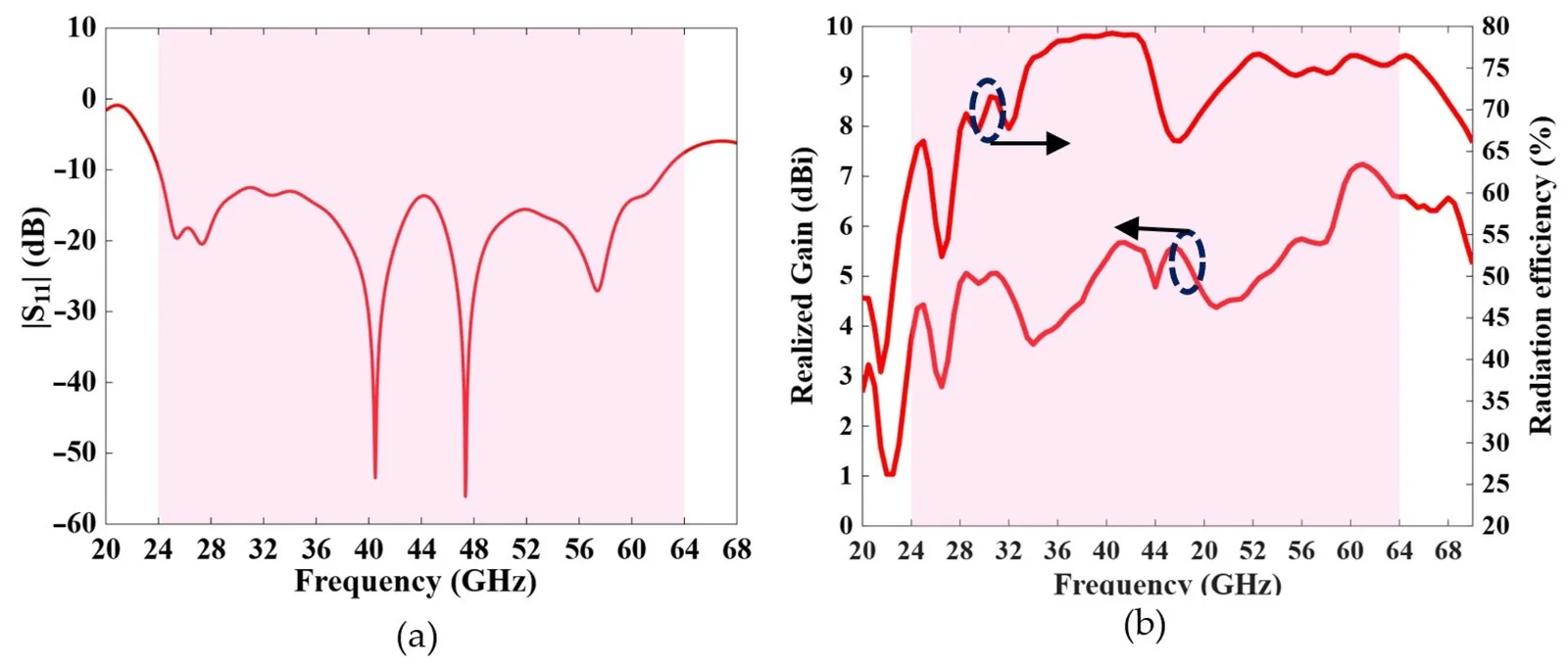
Simulated (**a**) reflection coefficient and (**b**) realized gain with radiation efficiency of the proposed four-port HSMA.

To further examine the radiation-performance behavior of the proposed four-port HSMA, Figure 11 presents the reflection coefficients, realized gain, and radiation efficiency over the operating band. The gain and efficiency responses are included before the surface-current analysis to identify the frequency regions where the radiation characteristics vary and to support the physical interpretation of these variations.

To explain the behavior observed in Figure 11, the simulated surface-current distribution is analyzed at representative frequencies selected from the reflection-coefficient, realized-gain, and radiation-efficiency responses. The selected frequencies represent the lower, middle, and upper regions of the super-wideband operating range, including frequencies where strong impedance matching and noticeable gain and efficiency variations are observed. Unlike the single-element analysis, the aim here is not only to explain the gain and efficiency behavior, but also to investigate current confinement and coupling between the MIMO ports.

**Figure 12:**
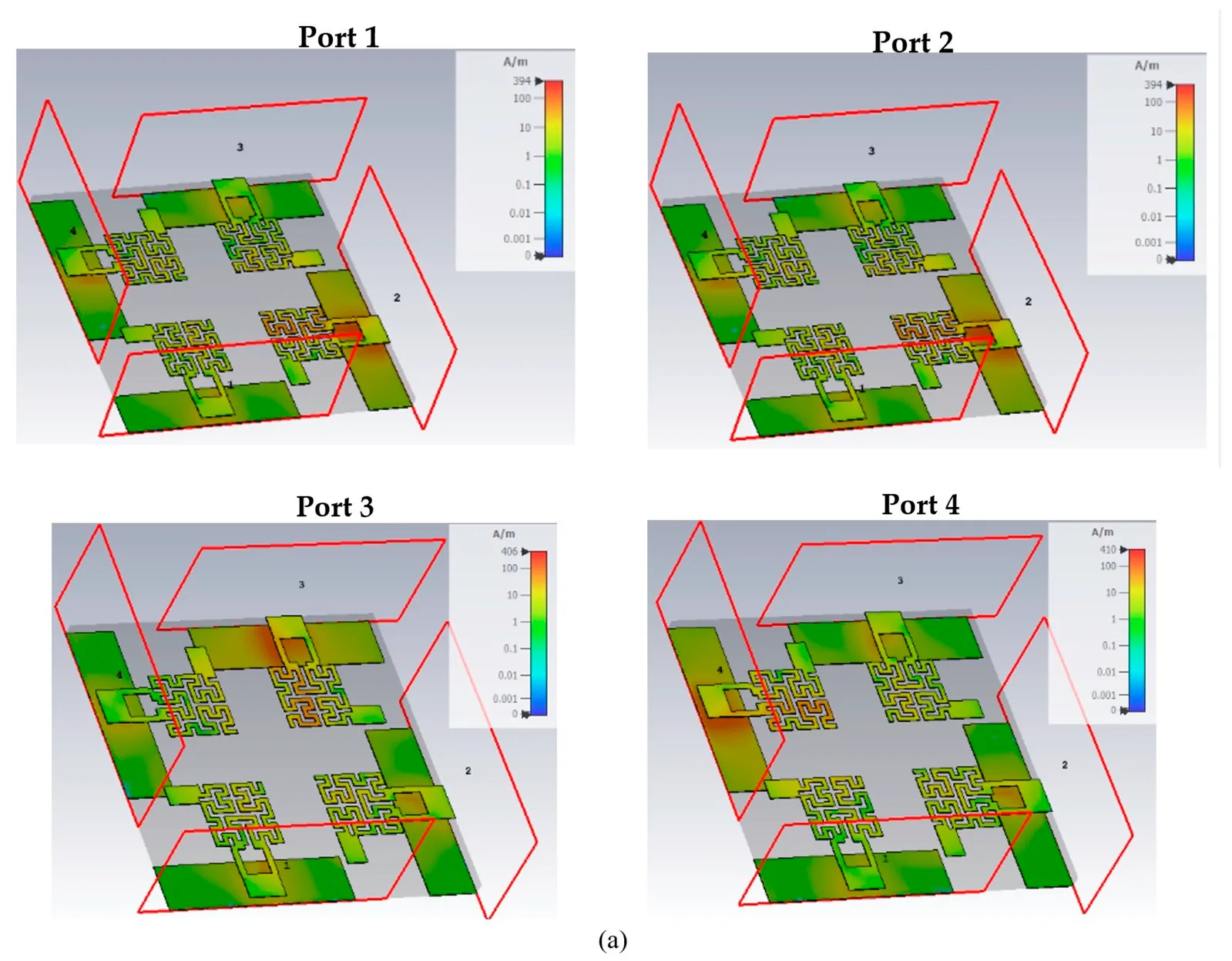

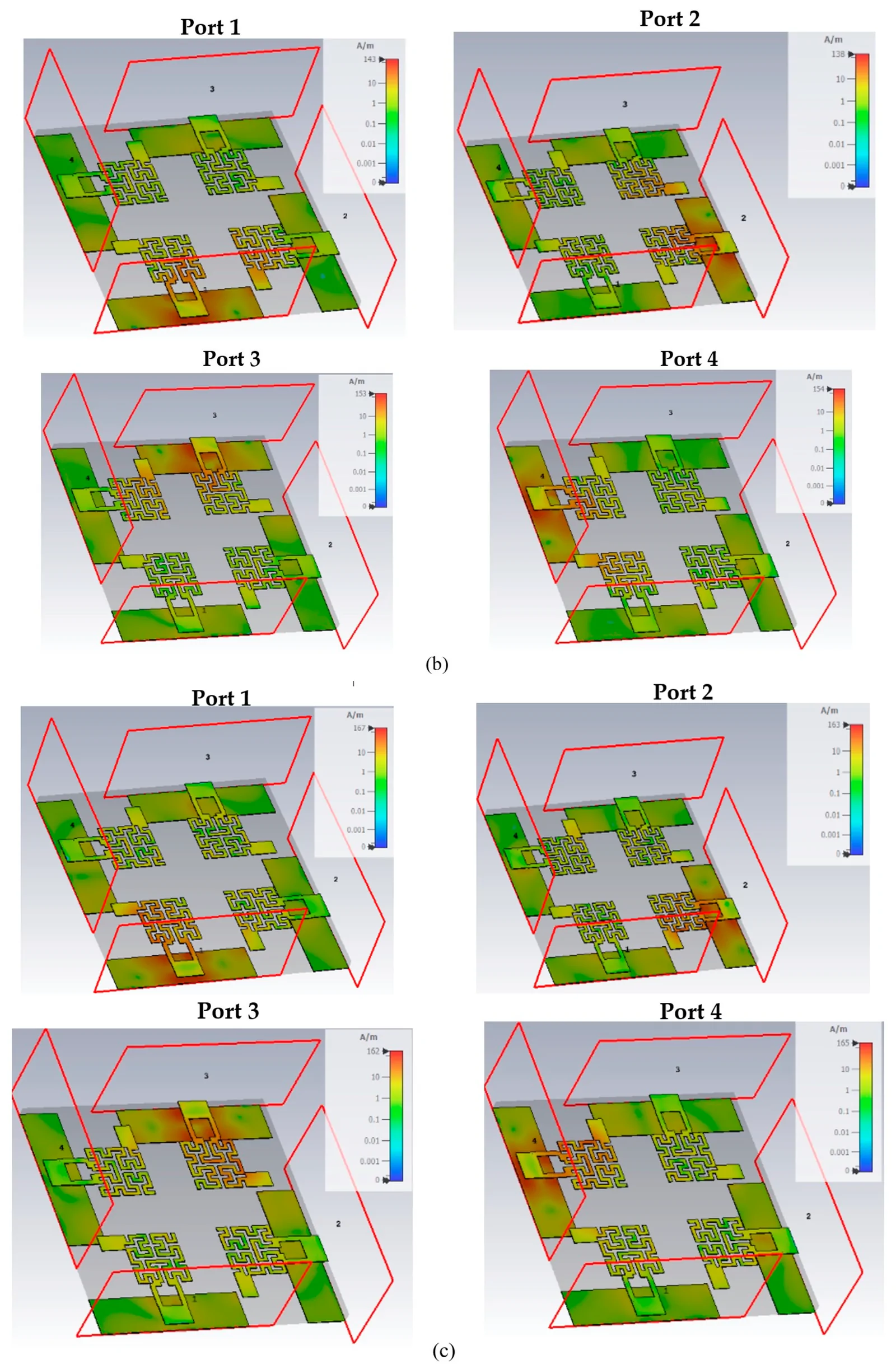

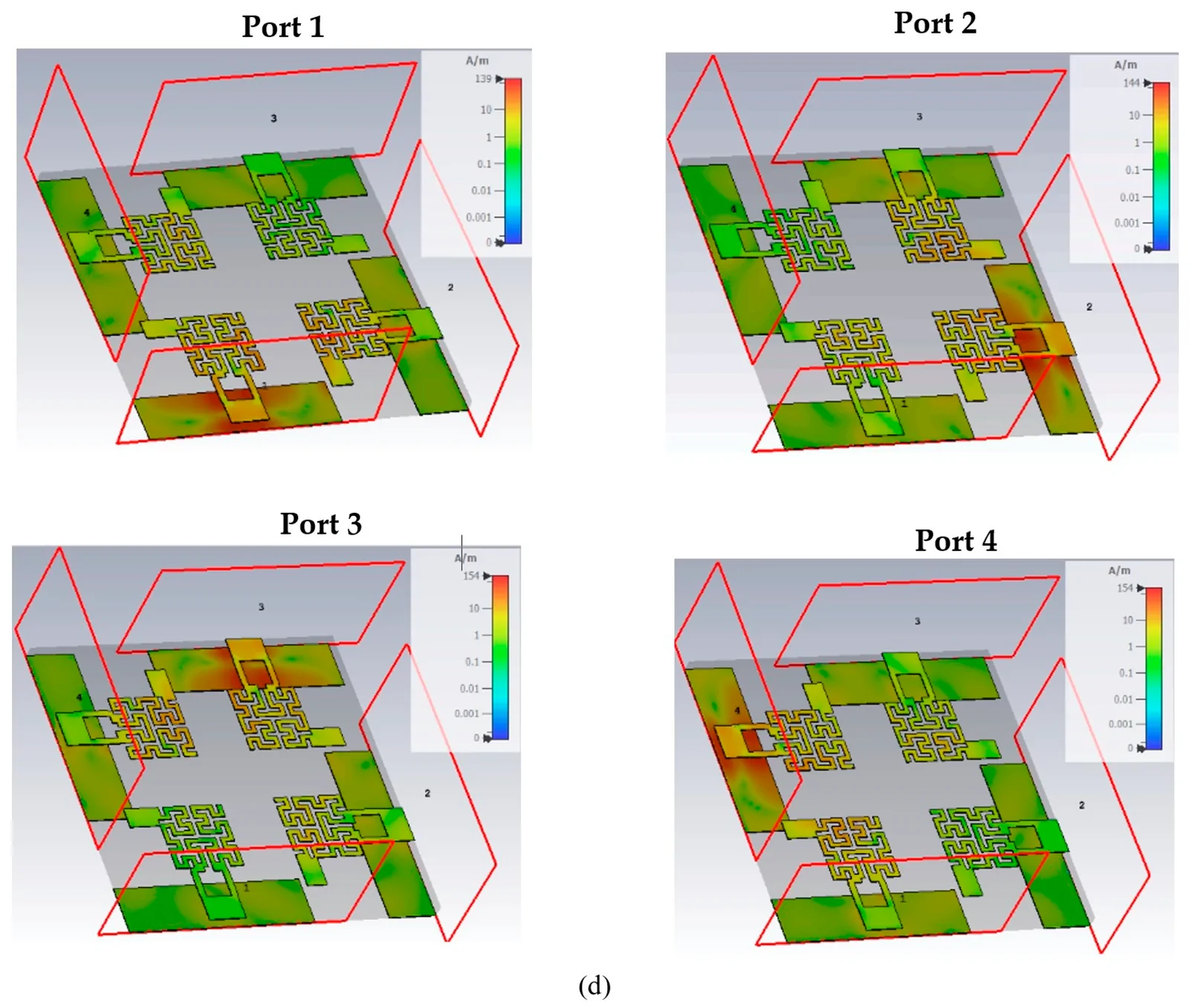
Simulated surface current distribution of the proposed four-port HSMA with one port excited and the remaining ports terminated with 50 Ω loads at: (**a**) 28 GHz, (**b**) 40 GHz, (**c**) 50GHz, and (**d**) 60 GHz.

Figure 12 shows the surface-current distribution of the proposed HSMA at frequencies: 28 GHz, 40 GHz, 50GHz, and 60 GHz when one port is excited, and the remaining ports are terminated with matched 50 Ω loads. In the lower-frequency region, the current is mainly concentrated around the feedline and the Hilbert-slot path of the excited element, confirming that the folded fractal geometry contributes to extending the effective current path. Only weak induced current is observed on the neighboring elements, indicating limited inter-port coupling. The relatively confined current distribution also explains the stable gain and radiation-efficiency behavior observed in Figure 11 at the lower part of the operating band.

At the mid-band resonant frequencies, where Figure 12 shows strong matching behavior, the current becomes more strongly distributed along the Hilbert-slot edges and the matching/extension regions of the excited element. This confirms that the optimized slot and extension sections support the formation of wideband resonances while maintaining compactness. Although small, induced currents appear on the adjacent elements, their magnitude remains lower than that on the excited port, which explains the good isolation behavior of the proposed HSMA. The smoother current transfer along the radiating structure also contributes to the improved gain and radiation-efficiency responses observed in Figure 11.

At the upper-frequency region, the current spreads over a larger portion of the Hilbert-slot structure and associated extensions. This wider current utilization supports the improved gain response observed in Figure 11. The current remains mainly confined to the excited element, while the passive ports show limited coupling. The radiation-efficiency behavior can also be related to the observed current distribution. Frequencies exhibiting more uniform current flow across the radiating structure generally maintain higher efficiency, whereas stronger current concentration around narrow slot sections, feed transitions, and coupling regions can introduce additional losses and produce local efficiency reductions.

Overall, the surface-current distributions confirm that the operation of the proposed HSMA is not only controlled by the feedline, but by the combined contribution of the Hilbert-slot path, matching section, and extension regions. The changes in current confinement across the selected frequencies explain the observed gain and radiation-efficiency variations, while the weak induced currents on neighboring elements confirm the low mutual coupling and good isolation performance of the proposed four-port HSMA. These features support the super-wideband impedance matching, gain stability, radiation efficiency, and isolation observed in Figure 11, making the antenna suitable for mmWave wearable telemetry applications.

### 2.2. Bending and On-Body Performance

**Figure 13:**
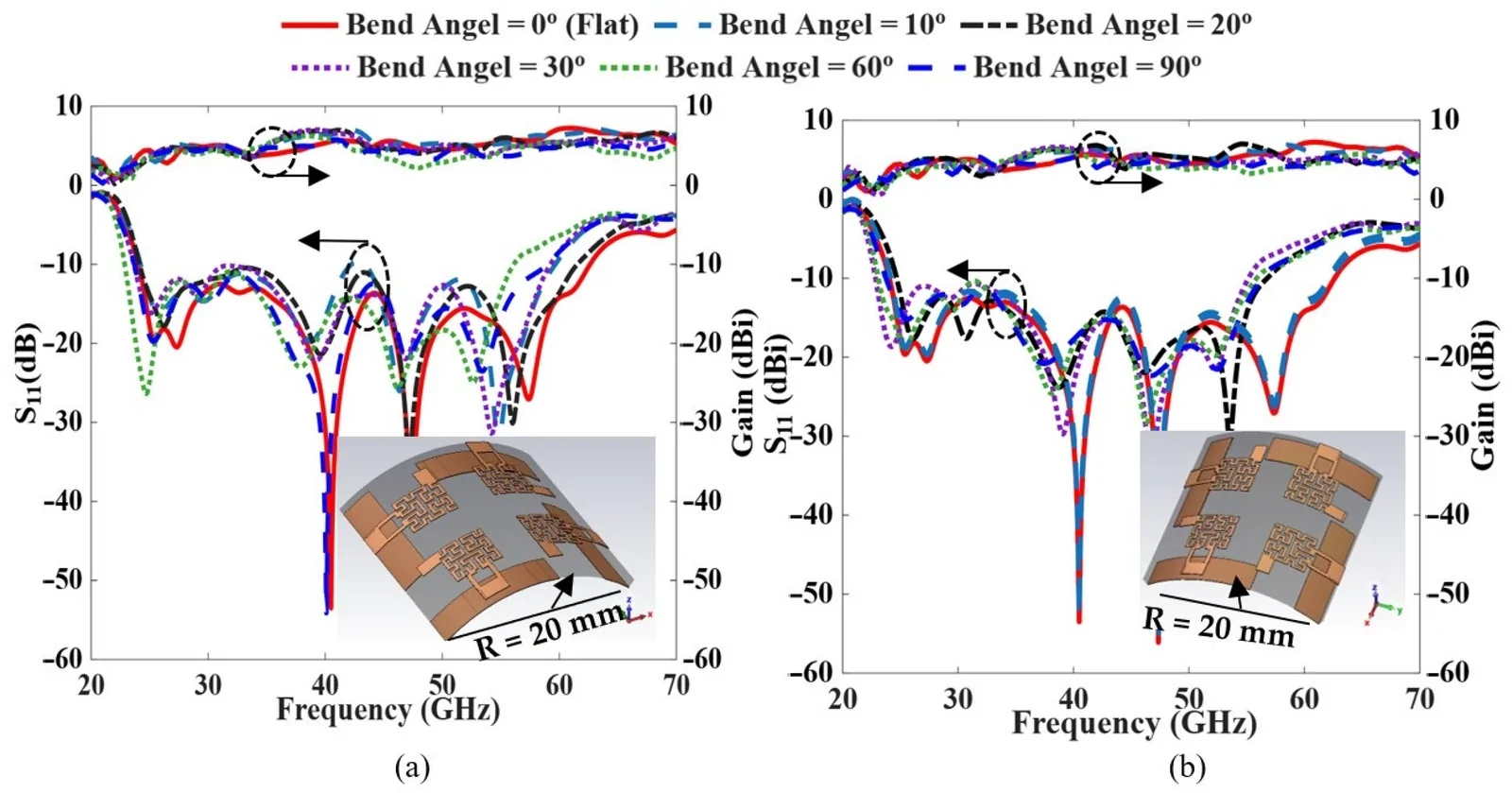
Simulated bending performance of the proposed four-port HSMA under different bend angles: 0° (flat), 10°, 20°, 30°, 60°, and 90°, showing the corresponding reflection coefficient and gain responses together with the bent antenna models: (**a**) x-axis and (**b**) y-axis.

Since the proposed four-port HSMA is intended for wearable medical telemetry, its bending performance is investigated to evaluate its robustness under mechanical deformation. The antenna is analyzed under two bending orientations: bending around the x-axis and bending around the y-axis, as shown in Figure 13a and Figure 13b, respectively. For each case, bend angles of 0° (flat), 10°, 20°, 30°, 60°, and 90° are considered.

The results of Figure 13a show that the reflection coefficient is close to the flat case for small and moderate bend angles. For 10°, 20° and 30°, we see only small shifts in frequency and the antenna is still well matched over a wide band over most of the FR2 operating band. Even under severe x-axis bending, the response remains acceptable. From Figure 13a, the 60° bent case maintains approximately S_11_ < −10 dB at 22.9–56.1 GHz, while the 90° bent case maintains approximately S_11_ < −10 dB at 23.65–59.6 GHz, with some local mismatch appearing near the upper edge of the band.

For bending around the y-axis, Figure 13b shows a more noticeable effect on the antenna response. Compared with x-axis bending, y-axis bending produces larger variations in both S11 and gain, especially for the 60° and 90° cases. The approximate matched bandwidth is still preserved over most of the operating range, but stronger local deviations are observed in the mid- and upper-frequency regions. From Figure 13b, the 60° bent case maintains approximately S_11_ < −10 dB at 23.15–56.25 GHz, while the 90° bent case maintains approximately S_11_ < −10 dB at 23.75–56.15 GHz, with more visible degradation at the upper band.

The gain response follows the same general trend as the flat case for both bending orientations, although moderate fluctuations occur as the bending angle increases. The variation in gain can be attributed to the change in the effective current path, the curvature of the antenna, and the radiation aperture during the mechanical deformation. The results suggest that the bending along the y-axis is the more critical bending condition for the proposed HSMA, as it results in a more significant shift in the reflection coefficient and the gain response. Nevertheless, the antenna maintains a wide matched response under both bending orientations, indicating its ability to be embedded in curved wearable medical devices.

### 2.3. On-Body Radiation Performance and Telemetry-Oriented Port Selection

**Figure 14:**
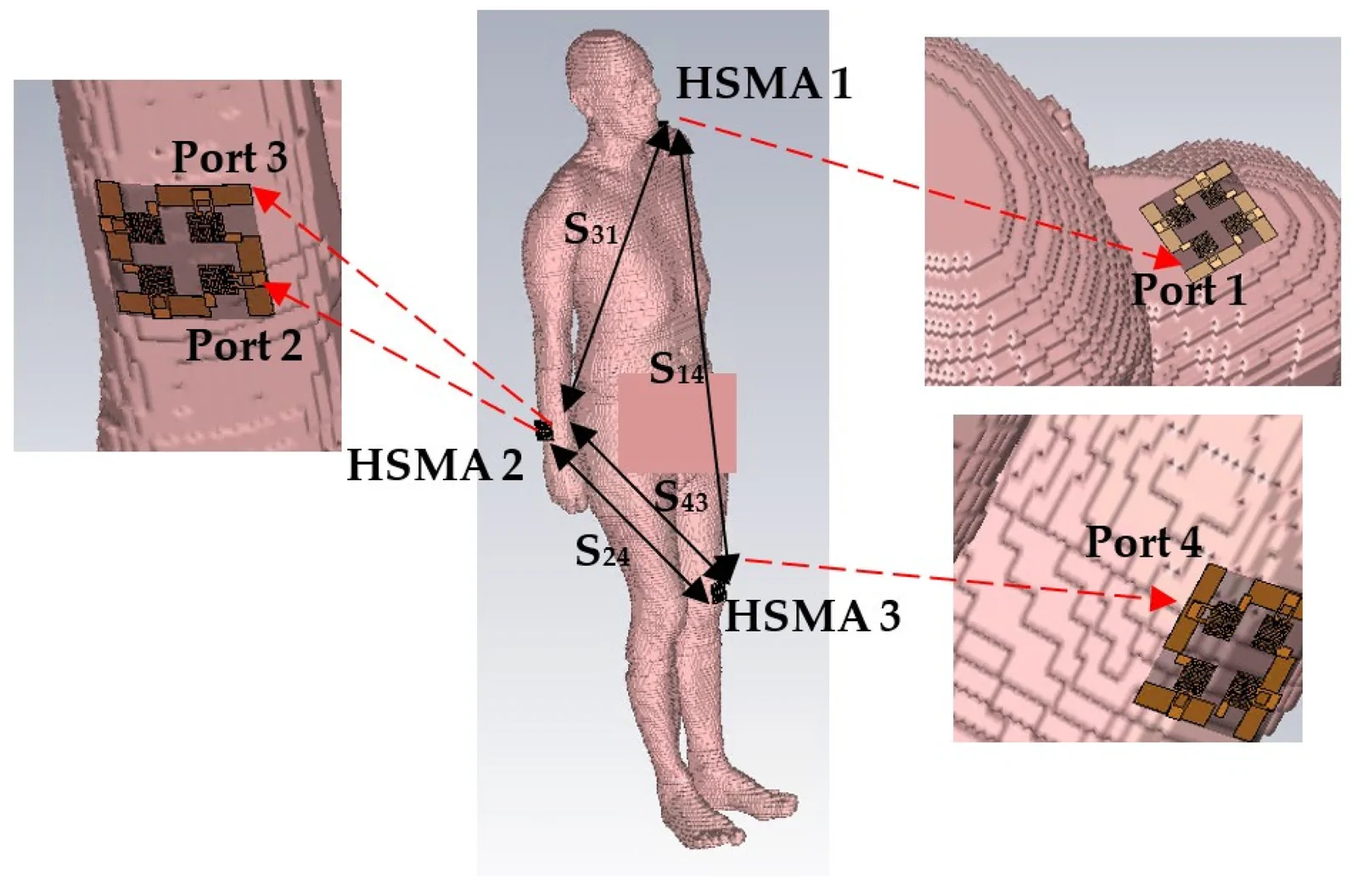
On-body placement of the proposed four-port HSMA on the CST Gustav voxel phantom for body-centric telemetry.

To evaluate the suitability of the proposed four-port HSMA for wearable mmWave telemetry, the antenna is placed directly on the CST Gustav voxel phantom, as shown in Figure 14. Three representative body locations are considered: knee, wrist, and shoulder. These locations are selected to emulate distributed wearable medical telemetry nodes positioned on different parts of the body. The wrist-mounted HSMA represents a smartwatch-type or compact medical monitoring device, while the knee- and shoulder-mounted HSMAs represent additional sensing or relay nodes in a body-centric telemetry system.

The antenna is placed directly on the CST Gustav voxel phantom at the selected shoulder, wrist, and knee locations. The wrist-mounted case is also bent by 30° to represent a realistic conformal wearable condition, such as integration into a smartwatch-type or wrist-worn medical monitoring device. This placement scenario allows the antenna performance to be evaluated under realistic body curvature, tissue loading, bending, and orientation-dependent propagation conditions.

The active ports are selected according to the physical orientation of the HSMA at each body location and the intended telemetry direction. The knee-mounted HSMA is selected to be port 4, which is facing the wrist-mounted node. For the wrist-mounted HSMA, both Port 2 and Port 3 are considered because the wrist device can support more than one telemetry direction. In one case, the wrist node communicates with the knee-mounted HSMA, while in another case it supports communication with the shoulder-mounted HSMA. For the shoulder-mounted HSMA, Port 1 is selected because it is oriented towards the lower-body direction and supports the intended shoulder-to-wrist or shoulder-to-knee telemetry paths. This orientation-based active-port selection is illustrated in Figure 14 and is used consistently for the on-body S-parameter, gain, radiation-pattern, and later transmission coefficient-based link analyses.

This configuration highlights one of the main advantages of using a compact MIMO antenna for wearable medical telemetry. Unlike a single-element antenna, the proposed four-port HSMA provides multiple possible radiation paths and port combinations. If one port becomes less effective due to body shadowing, detuning, posture variation, or unfavorable orientation, another port can provide an alternative communication path. This diversity improves the reliability of the body-centric link and supports continuous medical monitoring, where stable data transfer between body-worn devices is required. Therefore, the proposed HSMA is not only a compact radiating structure, but also an effective MIMO telemetry antenna that can be integrated into wearable medical devices for reliable and efficient body-centric communication.

The configuration is also relevant to emerging connected medical devices and smartwatch-type monitoring platforms, where compact wireless hardware is required to support continuous sensing, remote monitoring, and reliable patient data transfer. Commercial and clinical systems such as wearable multi-parameter monitors, connected cardiac monitoring platforms, and wireless medical telemetry services highlight the increasing need for reliable body-worn communication links in healthcare applications [19,20,21]. In addition, recent 5G healthcare and mmWave technology developments indicate growing interest in high-data-rate, low-latency wireless platforms for medical connectivity and remote patient monitoring [22].

**Figure 15:**
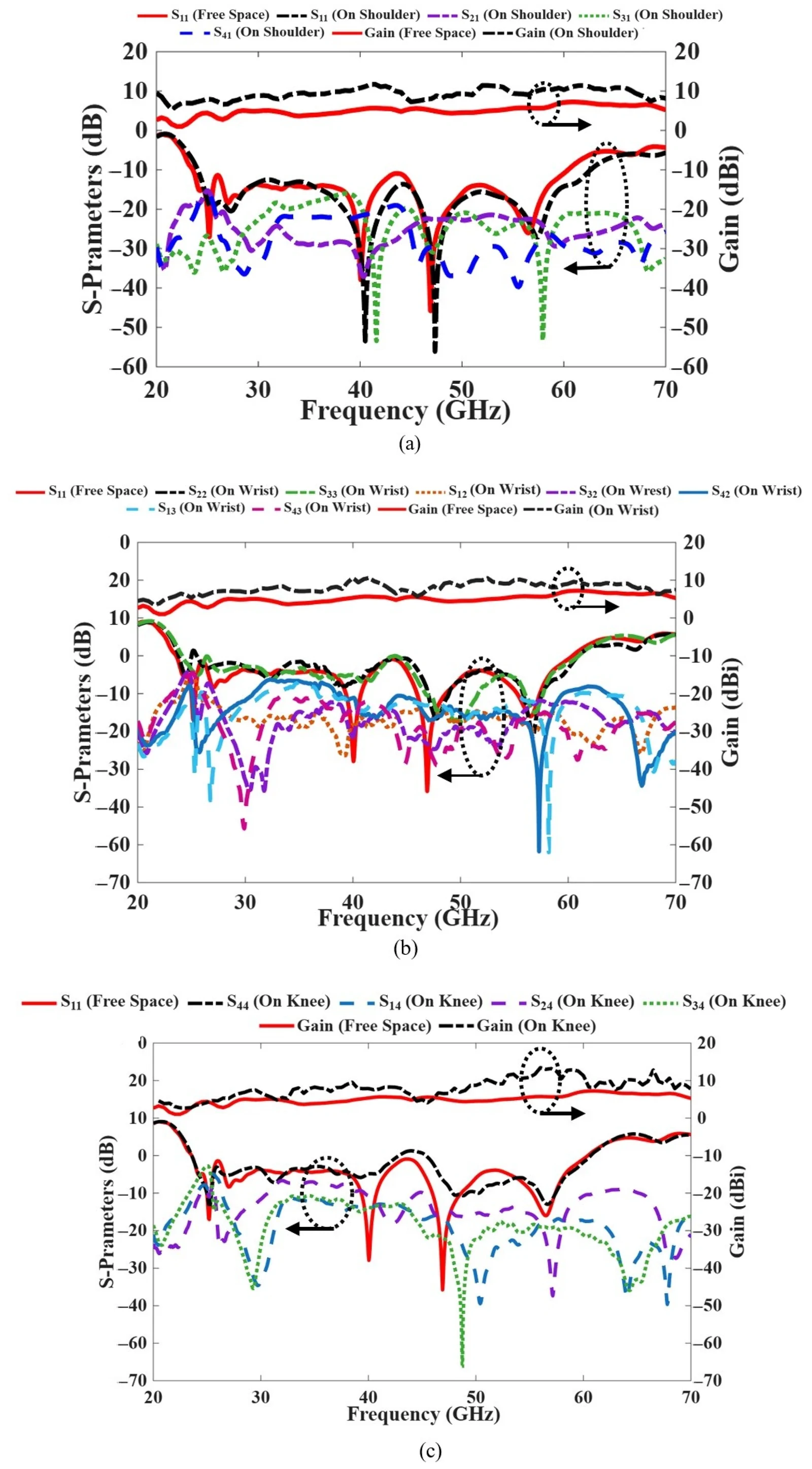
Comparison of the free-space and on-body S-parameters and realized gain of the proposed four-port HSMA on the CST Gustav voxel phantom: (**a**) shoulder placement, (**b**) wrist placement with 30° bending, and (**c**) knee placement.

The on-body S-parameters, inter-port isolation, and realized gain of the proposed HSMA are compared with the free-space response, as shown in Figure 15. In free space, the proposed four-port HSMA achieves super-wideband impedance matching from 24.05 to 62.64 GHz, corresponding to a bandwidth of 38.57 GHz. When the antenna is placed on the shoulder, the response remains close to the free-space case, with only small frequency shifts caused by tissue loading and body proximity. As shown in Figure 15a, the shoulder-mounted HSMA maintains an approximately matched response over about 24–63 GHz. The inter-port isolation also remains mostly better than 20 dB across the operating band, confirming that the MIMO performance is preserved when the antenna is mounted on the upper body.

For the wrist-mounted case, shown in Figure 15b, the body-loading effect is slightly more noticeable because the antenna is also bent by 30° to follow the wrist curvature. This condition represents a realistic conformal wearable scenario, such as integration into a smartwatch-type or wrist-worn medical monitoring device. The on-body response shows some local variation in the reflection coefficient and realized gain compared with the free-space case; however, the antenna still maintains a wide matched response over approximately 24–60 GHz. The isolation between the selected MIMO ports remains generally better than 20 dB over most of the operating band, indicating that the 30° wrist bending and tissue proximity do not severely degrade the MIMO behavior.

For the knee-mounted case, shown in Figure 15c, the antenna also maintains a wide impedance response compared with the free-space condition. The on-body response remains approximately matched from about 24–62 GHz, with some local shifts due to body curvature and the lossy dielectric properties of the voxel tissues. The isolation response remains acceptable and is generally better than 20 dB across most of the operating range, confirming that the compact four-port configuration can still provide low mutual coupling when mounted on the lower body.

The realized gain follows the same general trend as the free-space case for the three body placements, although some location-dependent variations are observed. These variations are mainly attributed to absorption, scattering, and changes in the radiation environment caused by the high-dielectric body tissues and different antenna orientations. Despite these effects, no severe gain degradation is observed, and the antenna maintains practical radiation performance for all investigated placements.

These results demonstrate that the proposed HSMA remains operational when mounted on different body regions of the CST Gustav voxel phantom. Although the on-body cases introduce some frequency shifts, gain fluctuations, and local variations in the S-parameters, the antenna continues to provide super-wideband operation and inter-port isolation, mostly better than 20 dB. This confirms the suitability of the proposed compact HSMA for distributed wearable medical telemetry, where the antenna may be integrated into wrist-worn, knee-mounted, or shoulder-mounted monitoring nodes.

Following the on-body S-parameter and gain analysis, the radiation characteristics of the proposed four-port HSMA are examined to further assess its suitability for body-centric mmWave telemetry. Representative 2D and 3D radiation patterns are presented at selected frequencies across the operating band to avoid excessive repetition while still demonstrating the radiation behavior under realistic body placement. The selected frequencies of 28, 40, 50, and 60 GHz represent the lower, middle, upper-mid, and upper regions of the super-wideband response. The 3D patterns in Table 2 show that the proposed HSMA mainly radiates outward from the body surface for the investigated placements, which is desirable for wearable telemetry because it supports communication between distributed body-worn nodes while reducing unnecessary radiation into lossy tissues. The corresponding 2D patterns confirm that practical radiation behavior is maintained for the selected body locations and active ports, although some variation is observed due to body curvature, tissue loading, and antenna orientation.

The radiation-pattern results confirm that the proposed HSMA can maintain suitable body-loaded radiation performance when placed at different body locations. However, the radiation patterns alone are not used to confirm the complete telemetry-link performance. Instead, they demonstrate that the antenna provides suitable radiation behavior for body-centric communication, while the quantitative link feasibility is evaluated in the following Section 2.4 using CST-simulated S_21_, received power, and link margin.

### 2.4. SAR Calculation

To verify the electromagnetic safety of the proposed four-port HSMA for wearable medical telemetry, the specific absorption rate (SAR) is evaluated using the CST Gustav voxel phantom. The SAR analysis is performed for the same body locations and telemetry-oriented active ports defined in Figure 14. Therefore, Port 4 is excited for the knee-mounted HSMA, Ports 2 and 3 are considered for the wrist-mounted HSMA, and Port 1 is excited for the shoulder-mounted HSMA. The wrist-mounted case is bent by 30° to represent a realistic conformal wearable condition, such as integration into a smartwatch-type or wrist-worn medical monitoring device.

The SAR values are calculated at the same representative frequencies used for the on-body radiation-pattern analysis, namely 28, 40, 50, and 60 GHz. These frequencies cover the lower, middle, upper-mid, and upper regions of the proposed antenna’s super-wideband operating range. For each case, the SAR is evaluated over both 1 g and 10 g of biological tissue using an input power of 100 mW (20 dBm). This allows the safety performance of the antenna to be assessed under realistic body-loading conditions and under the same port-orientation scenario used for the radiation-pattern and telemetry-link analyses.

**Figure 16:**
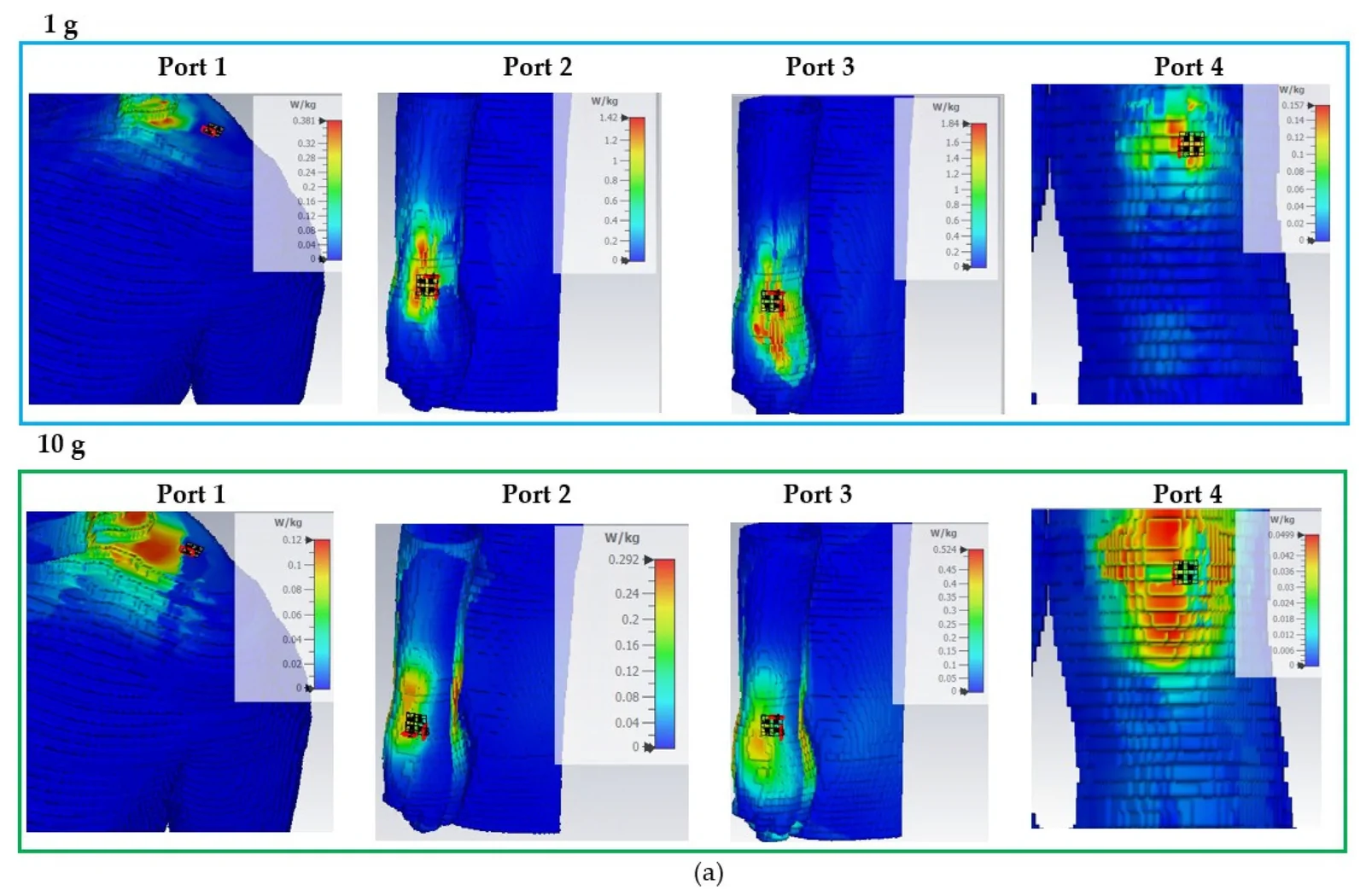

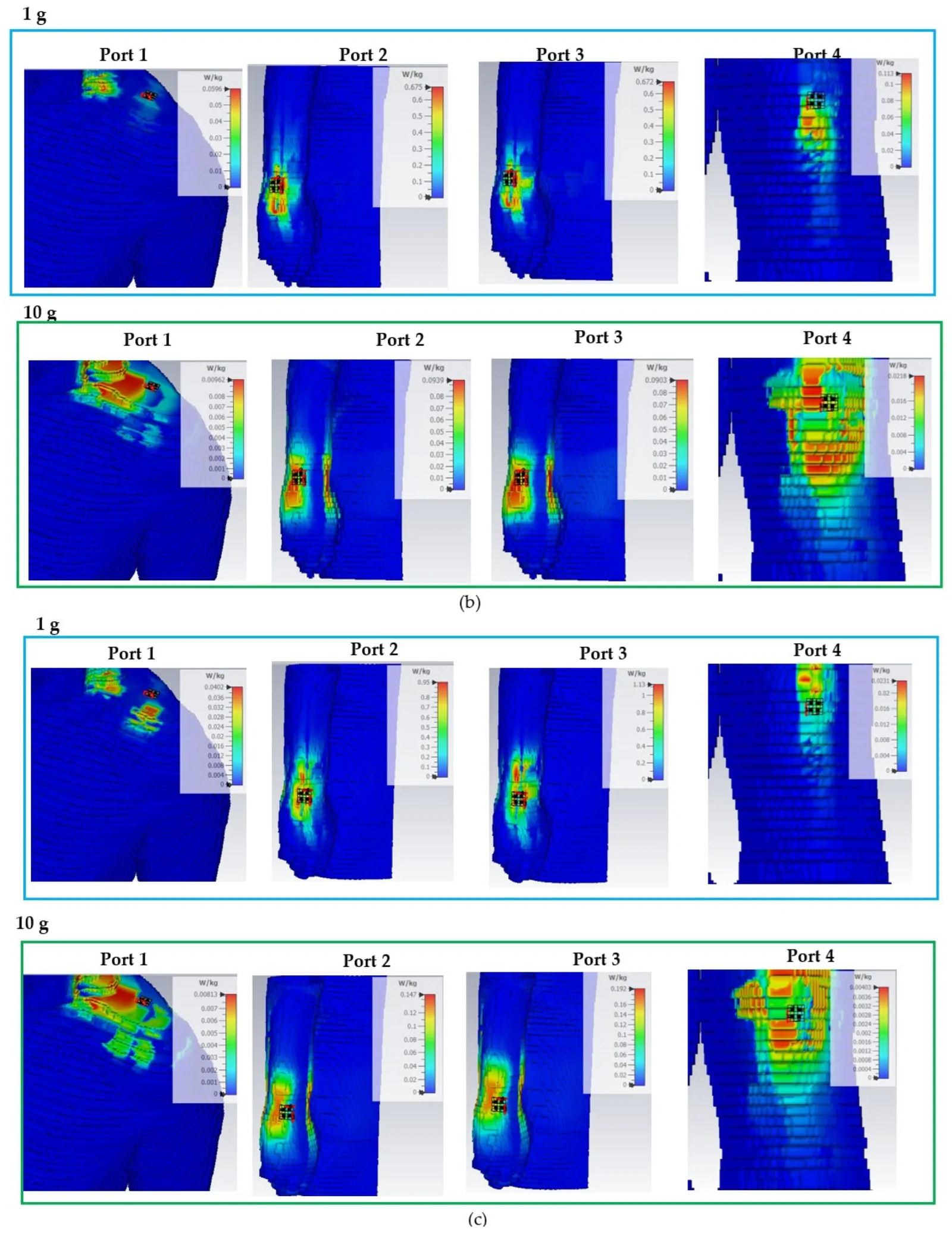

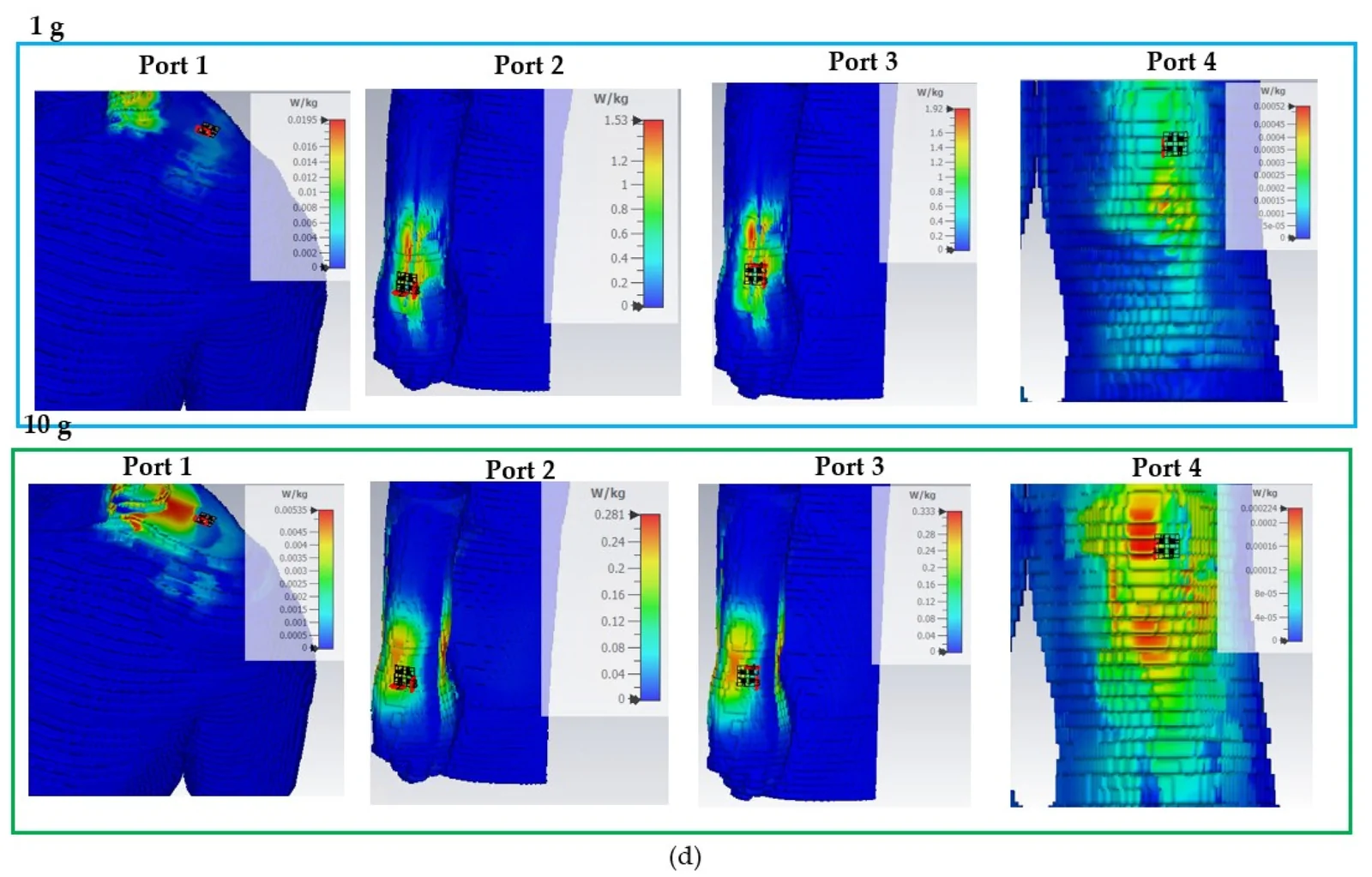
1 g and 10 g SAR values for the proposed compact HSMA on the shoulder (Port 1), wrist (Port 2 and Port 3) and knee (Port 4) of the CST-based Gustav phantom body model at (**a**) 28 GHz, (**b**) 40 GHz, (**c**) 50 GHz, and (**d**) 60 GHz.

Figure 16 shows the simulated SAR distributions of the proposed HSMA on the CST Gustav voxel phantom. The SAR hot spots are mainly localized around the antenna placement region and decrease rapidly away from the radiating structure. This behavior is expected at mmWave frequencies because the electromagnetic energy is strongly confined near the body surface due to the lossy and high-dielectric nature of human tissues. The results also show that the SAR distribution depends on the antenna location, port orientation, frequency, and local body curvature. For example, the wrist-mounted case is influenced not only by tissue loading but also by the 30° bending condition, while the knee and shoulder cases are mainly affected by local body curvature and antenna orientation.

For the knee-mounted HSMA, the SAR values are obtained when Port 4 is excited, corresponding to the telemetry-oriented path towards the wrist-mounted node. For the wrist-mounted HSMA, SAR is evaluated for both Port 2 and Port 3 because the wrist device can support more than one body-centric communication direction, including the knee-to-wrist and shoulder-to-wrist paths. For the shoulder-mounted HSMA, Port 1 is exciting because it is oriented towards the lower-body direction. This port-specific SAR evaluation is important because different ports may produce different local field distributions depending on their orientation relative to the body surface.

### 2.5. Application Scope: S-Parameter-Based Body-Centric Telemetry Link Performance

Following the on-body S-parameter, gain, SAR, and radiation-pattern analyses, the practical telemetry performance of the proposed four-port HSMA is evaluated using CST-simulated transmission coefficients [6]. The link analysis is based on the same orientation-based active-port configuration defined in Figure 14. In this configuration, the wrist-mounted HSMA represents a smartwatch-type or compact medical monitoring device, while the knee- and shoulder-mounted HSMAs represent distributed sensing or relay nodes in body-centric wearable telemetry system conditions [23]. This configuration is relevant to wearable medical monitoring scenarios where information from different body regions must be transferred reliably to a wrist-mounted device for processing, storage, or forwarding to an external hub. For example, the knee-mounted node may support gait, rehabilitation, joint-movement, or lower-limb activity monitoring, while the shoulder-mounted node may support posture, upper-body movement, rehabilitation tracking, or activity monitoring. These examples are considered as possible telemetry use cases, where the antenna system is responsible for reliable wireless data transfer rather than clinical diagnosis.

The selected telemetry paths are determined by the physical orientation of the active ports on the body. For the knee-to-wrist link, Port 4 of the knee-mounted HSMA 3 is used because it is oriented towards the wrist-mounted monitoring node. At the wrist (HSMA 2), both Port 2 and Port 3 are considered because the wrist-mounted device can support more than one telemetry direction. Therefore, the knee-to-wrist communication paths are evaluated using S_24_ and S_34_, where Port 4 is the transmitting port on the knee, and Ports 2 and 3 are the receiving ports on the wrist.

For the shoulder-to-wrist link, Port 1 of the shoulder-mounted HSMA 1 is used because it is oriented towards the lower-body/wrist direction. The corresponding shoulder-to-wrist communication paths are evaluated using S_21_ and S_31_, where Port 1 is the transmitting port on the shoulder, and Ports 2 and 3 are the receiving ports on the wrist. In addition, a direct shoulder-to-knee path may be considered using the corresponding selected ports, such as S_41_, if direct communication between the shoulder and knee nodes is required. The reverse paths, such as S_13_ or S_14_, may also be evaluated depending on the intended transmitting and receiving direction in the telemetry system.

This link-selection strategy highlights the advantage of using a compact MIMO antenna instead of a single-element wearable antenna. In a practical body-centric environment, one port may experience reduced performance because of body shadowing, detuning, bending, posture variation, wrist rotation, or an unfavorable propagation direction. In such cases, another port can provide an alternative communication path with lower path loss or higher received power. For example, if the knee-to-wrist path through Port 2 becomes weak, the system can use the knee-to-wrist path through Port 3. Similarly, the shoulder-to-wrist link can be evaluated through both wrist-facing ports. This diversity capability improves the reliability of the wearable telemetry link and supports continuous monitoring where stable data transfer between body-worn nodes is required.

The CST-simulated transmission coefficient *S_ij_* is used as the main parameter for the link analysis, where j denotes the transmitting port and i denotes the receiving port. Since *S_ij_* represents the electromagnetic power transfer between the selected body-mounted antennas, higher *S_ij_* values indicate stronger coupling and better telemetry link performance. This approach is particularly suitable for wearable mmWave telemetry because the simulated *S_ij_* response inherently includes the effects of antenna mismatch, realized gain, human-body absorption, tissue loading, near-field coupling, polarization mismatch, body shadowing, orientation, and multipath interaction. Therefore, the CST-simulated *S_ij_* parameter provides a full-wave electromagnetic measure of the coupling between the selected body-mounted antennas.

The simulated path loss is extracted directly from the transmission coefficient as

(1) $$PL_{\mathrm{sim}}\left(dB\right)=-S_{ij}\left(dB\right)$$

where a lower path-loss value indicates stronger electromagnetic coupling and a more favorable telemetry path between the selected body-mounted antennas. When the transmit power is known, the received power can be estimated from the CST-based transmission coefficient as

(2) $$P_{r,\mathrm{CST}}\left(dBm\right)=P_{t}\left(dBm\right)-PL_{\mathrm{sim}}\left(dB\right)$$

In this CST-based calculation, the antenna gain should not be added separately because the simulated port-to-port transmission coefficient already includes the antenna contribution, mismatch, body loading, and propagation effects. Adding the antenna gains again would result in double counting. Therefore, the CST-based received-power calculation is considered the primary method for evaluating the proposed body-centric telemetry links.

**Figure 17:**
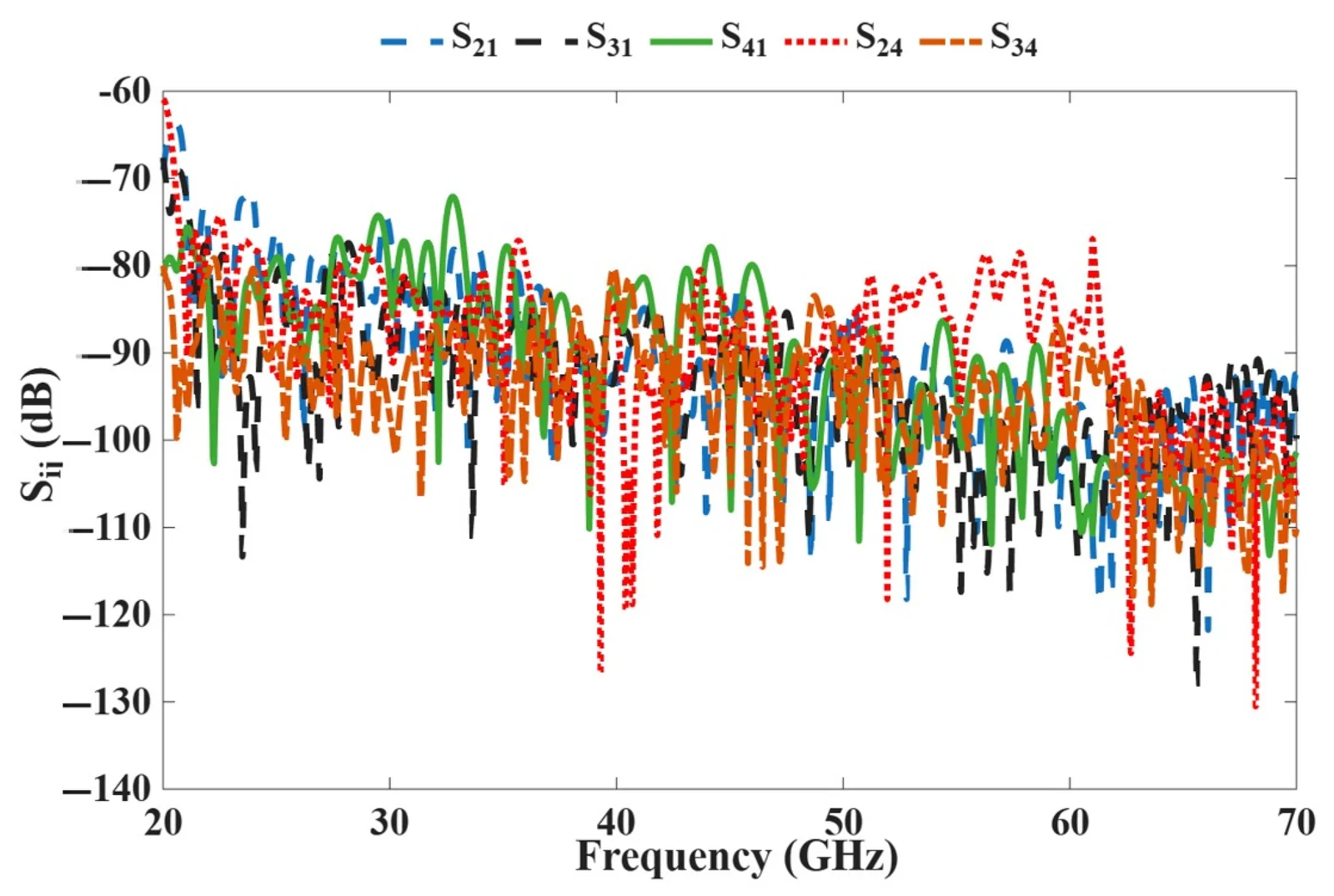
CST-simulated transmission coefficients for the selected body-centric telemetry paths using the orientation-based port configuration on the CST Gustav voxel phantom.

Figure 17 shows the CST-simulated link transmission characteristics (*S_ij_*) for the selected body-centric telemetry paths. The curves exhibit strong frequency-dependent behavior, which is expected for mmWave on-body propagation because the channel is affected by tissue absorption, body shadowing, antenna orientation, and multipath interaction around the voxel phantom. The corresponding simulated path losses range from approximately 75 dB to 110 dB across the operating band, with local maxima and fading notches depending on the selected port combination.

Among the knee-to-wrist links, S_24_ generally provides the stronger response over several parts of the band, particularly in the upper-frequency region around 50–62 GHz, where it remains closer to −80 dB compared with the other paths. This indicates that the Port 4-to-Port 2 orientation is favorable for knee-to-wrist telemetry in these frequency regions. The S_34_ response also provides an alternative knee-to-wrist path, but it is generally more fluctuating and slightly weaker than S_24_ over much of the band. Therefore, S_34_ can be considered as a diversity/backup path when _S24_ experiences local fading.

For the shoulder-to-wrist links, S_21_ provides stronger coupling at the lower part of the band, especially close to 20–25 GHz, while S_31_ is generally weaker and shows deeper fading at several frequencies. This suggests that the Port 1-to-Port 2 path is more favorable than the Port 1-to-Port 3 path for shoulder-to-wrist telemetry under the selected body orientation. However, S_31_ remains useful as an alternative MIMO path because it may become favorable under different body posture, wrist rotation, or antenna orientation.

The S_41_ response, representing the direct shoulder-to-knee path, shows relatively strong coupling in some lower- and mid-band regions, particularly around 28–34 GHz. This indicates that a direct upper-body to lower-body link can also be supported when required. However, for the proposed monitoring scenario, the main intended paths are the knee-to-wrist and shoulder-to-wrist links, since the wrist-mounted HSMA acts as the central wearable monitoring node.

These results demonstrate the importance of combining MIMO operation with super-wideband performance for wearable telemetry. No single port combination provides the strongest response across the entire operating band. Instead, different links become favorable at different frequencies because of body-induced fading, orientation-dependent propagation, tissue loading, and local shadowing. This behavior highlights the advantage of the proposed wideband HSMA, since the system is not restricted to a single narrow operating band. Instead, several effective frequency regions can be identified across the wide operating bandwidth, allowing the telemetry system to select the frequency range and port combination that provide the highest transmission coefficient or lowest path loss. Therefore, the proposed four-port HSMA provides both spatial diversity through its multiple ports and frequency diversity through its super-wideband response. This is particularly important for continuous medical monitoring, where reliable data transfer must be maintained despite body movement, posture change, wrist rotation, and local body shadowing.

To assess whether the received signal is sufficient for reliable communication, the link margin is calculated by comparing the received power with the receiver sensitivity. The receiver’s sensitivity is obtained as

(3) $$P_{sens}\left(dBm\right)=-174+10\log_{10}\left(B\right)+NF+{SNR}_{req}$$

where B is the channel bandwidth in Hz, NF is the noise receiver figure, and SNR_req_ is the required signal-to-noise ratio. The link margin is then calculated as

(4) $$LM\left(dB\right)=Pr,CST\left(dBm\right)-P_{sens}\left(dBm\right)$$

A positive link margin indicates that the received signal is higher than the receiver sensitivity and that the selected telemetry path can support communication under the assumed system parameters.

Two representative telemetry assumptions are considered. The first assumption represents a low-power wearable telemetry case with $P_{t}$ = **0 dBm**, B = **100 MHz**, NF = **6 dB**, **and** ${SNR}_{req}$ = **10 dB**. This gives a receiver sensitivity of approximately **−78 dBm**. The second assumption represents a higher-rate short-range mmWave telemetry case with $P_{t}$ = **10 dBm**, B = **1 GHz**, NF = **4 dB**, **and** ${SNR}_{req}$ = **6 dB**. This gives a receiver sensitivity of approximately **−74 dBm.** Based on these values, the link margin can be directly obtained from the CST-simulated S_ij_ curves. For the first assumption, the link margin becomes

(5) $$LM=S_{ij}+78\;dB$$

while for the second assumption it becomes

(6) $$LM=S_{ij}+84\;dB$$

**Figure 18:**
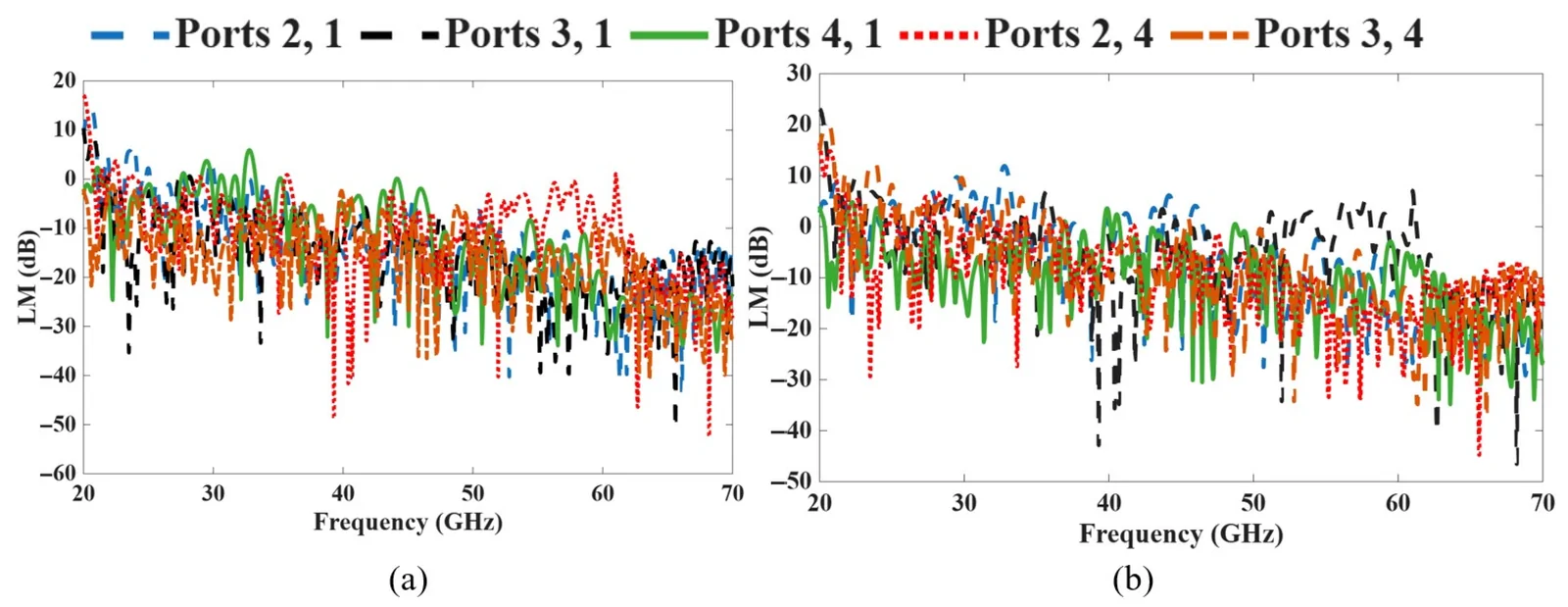
Calculated link margin versus frequency for the selected body-centric telemetry paths: (**a**) first assumption; and (**b**) second assumption.

Figure 18 presents the calculated link margin for the selected body-centric telemetry paths under the first and second assumptions. The link-margin curves follow the same frequency-dependent behavior observed in the CST-simulated transmission coefficients, with local peaks and fading notches caused by body absorption, tissue loading, antenna orientation, and multipath interaction around the CST Gustav voxel phantom.

Under the first assumption, positive link margin is mainly achieved in the frequency regions where the transmission coefficient is relatively strong. The S_24_ path, corresponding to the knee-to-wrist link from Port 4 to Port 2, provides the highest peak link margin and therefore represents the most efficient knee-to-wrist telemetry path. The S_21_ path also achieves positive link margin in several lower-frequency regions, indicating that the shoulder-to-wrist link through Port 2 can support telemetry under favorable channel conditions. The S_41_ path provides useful additional coupling in some lower- and mid-band regions, while S_34_ is generally weaker and acts mainly as a diversity or backup path.

Under the second assumption, the link margin improves because of the higher transmit power. More frequency regions show positive or less negative link margin, especially for S_24_, S_21_, and S_41_. This confirms that the proposed HSMA can support higher-rate short-range wearable telemetry when sufficient transmit power is available. However, the link margin remains strongly dependent on frequency and port combination, confirming that no single path provides the best performance across the entire operating band.

The link-margin results confirm that S_24_ is the most suitable primary knee-to-wrist link, while S_21_ is the most suitable primary shoulder-to-wrist link under the selected body orientation. The S_34_ and S_31_ paths are less efficient on average but remain valuable as diversity links when the primary paths are affected by fading, posture variation, or local body shadowing. The S_41_ path can support direct shoulder-to-knee communication in selected frequency regions, but it is not the main telemetry route for the proposed wrist-centered monitoring configuration.

The S-parameter-based link analysis complements the on-body radiation-pattern results discussed earlier. The radiation patterns demonstrate that the selected ports provide suitable outward radiation behavior from the body surface, while the S_ij_-based received-power and link-margin calculations quantify the practical communication feasibility between the distributed wearable nodes. These results confirm that the proposed compact four-port HSMA can support efficient and reliable mmWave telemetry between knee-, wrist-, and shoulder-mounted medical or monitoring devices.

## 3. Experimental Validation and Discussion of Results

**Figure 19:**
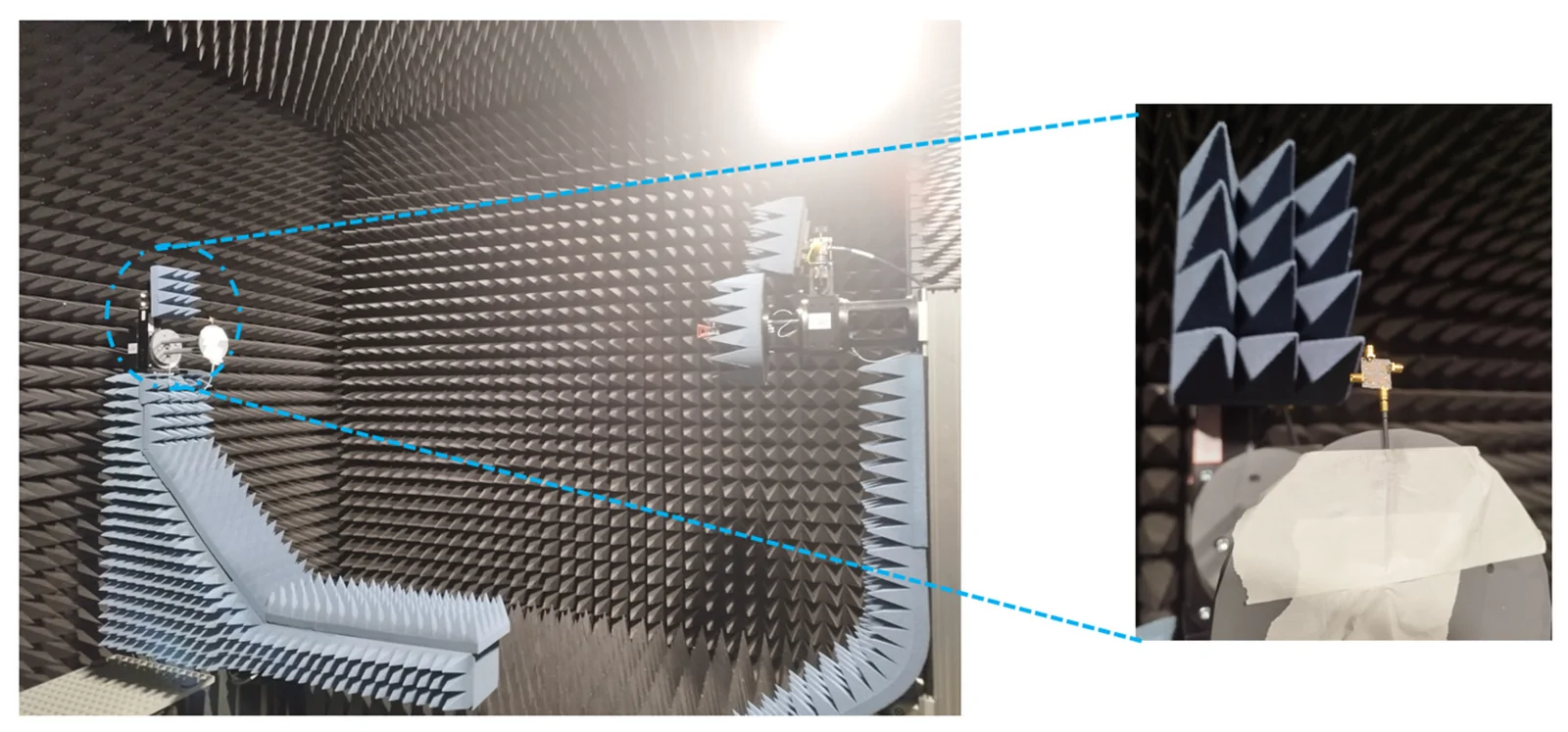
Measurement setup at the NSI-AMETEK anechoic chamber.

The proposed four-port HSMA was fabricated using the LPKF ProtoMat S104 PCB milling system. The reflection coefficients were measured using a Keysight PNA-X vector network analyzer, while the radiation patterns and realized gain were measured using the NSI/AMETEK anechoic chamber system at WiSAR Lab, ATU. The chamber provides an automated spherical near-field antenna measurement for radiation characterization up to 50 GHz. Figure 19 shows the measurement environment and a close-up view of the fabricated HSMA mounted during the chamber measurement. During the chamber measurement, the fabricated HSMA was connected through a 1.85 mm coaxial connector, and the measured response was used to obtain the far-field radiation characteristics.

**Figure 20:**
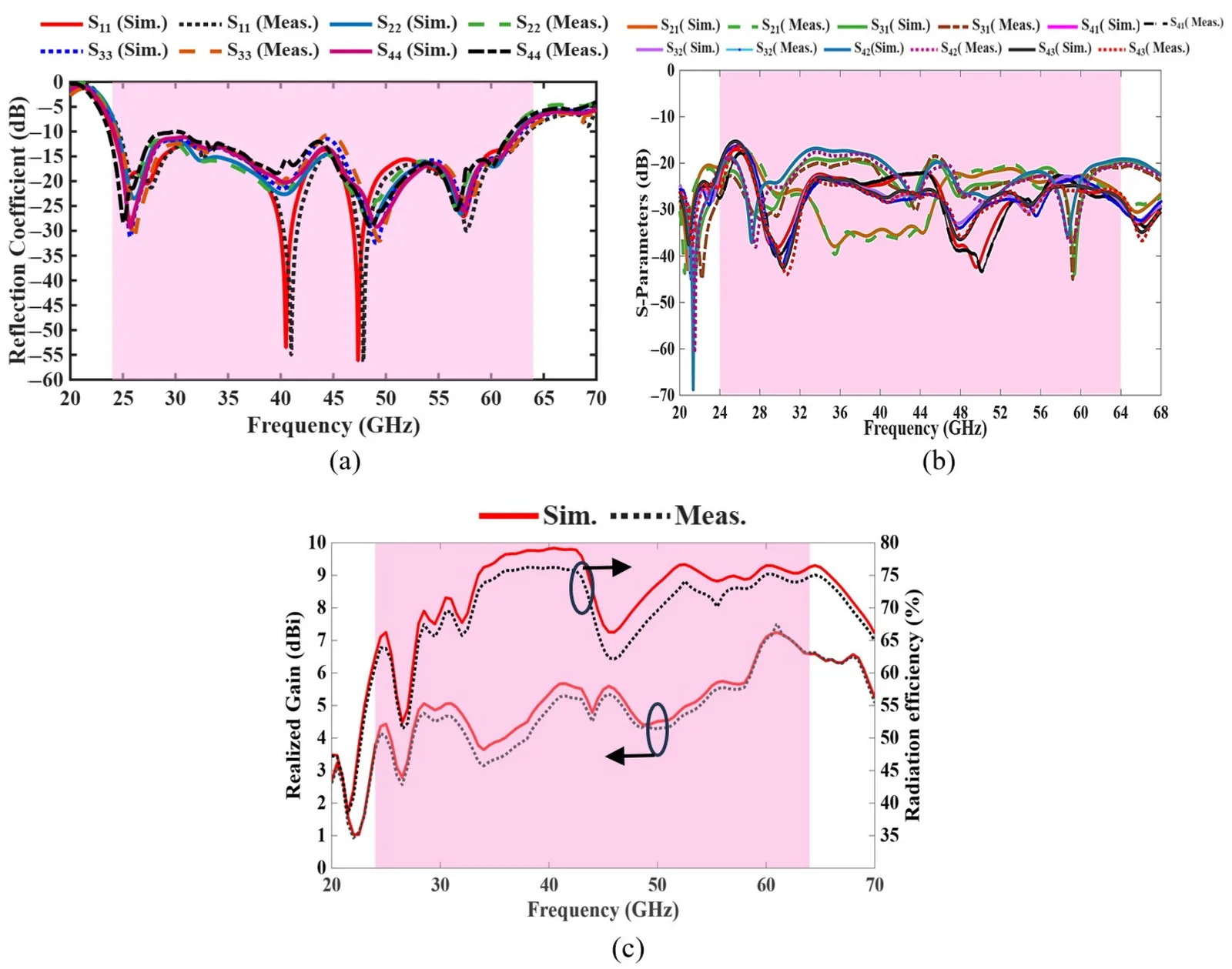
Simulated and measured performance of the proposed four-port HSMA: (**a**) reflection coefficients, (**b**) inter-port transmission coefficients, and (**c**) realized gain and radiation efficiency.

Figure 20 presents the simulated and measured performance of the proposed four-port HSMA in terms of impedance matching, inter-port coupling, realized gain, and radiation efficiency. Figure 20a shows the reflection-coefficient responses of the four ports, including S_11_, S_22_, S_33_, and S_44_. The results confirm that the fabricated HSMA maintains wide impedance matching across the highlighted mmWave operating band. For the representative S_11_ response, the simulated result remains below −11.34 dB from 24.66 to 62.59 GHz, corresponding to a bandwidth of 37.93 GHz, while the measured result remains below −11.86 dB from 24.71 to 63.81 GHz, corresponding to a bandwidth of 39.1 GHz. The measured reflection coefficients follow the simulated responses with small frequency shifts and amplitude variations, which verifies the practical realization of the compact four-port HSMA and confirms that the fabricated prototype preserves the intended wideband behavior.

Figure 20b presents the simulated and measured inter-port transmission coefficients, including S_21_, S_31_, S_41_, S_32_, S_42_, and S_43_, which are used to evaluate the MIMO isolation performance. As shown in the figure, the coupling levels remain mostly below −20 dB across the main operating band, indicating good isolation between the closely spaced ports. This low mutual coupling confirms that the compact four-port arrangement can support MIMO operation without severe interaction between the antenna elements. The small differences between the simulated and measured transmission-coefficient curves are expected at mmWave frequencies and can be attributed to fabrication tolerances, substrate-property variation, connector alignment, soldering effects, cable losses, calibration uncertainty, and antenna positioning during measurement.

Figure 20c compares the simulated and measured realized gain and radiation efficiency of the proposed HSMA over the highlighted operating band of 24–64 GHz. The realized gain follows the same overall trend in both simulation and measurement, with the measured response remaining close to the simulated curve across the band. From the plotted response, the simulated realized gain varies approximately from 2.79 to 7.24 dBi, while the measured realized gain varies approximately from 2.57 to 7.52 dBi, with the peak measured value observed near the upper part of the band. The simulated radiation efficiency remains approximately within 52.35–79.17%, while the measured radiation efficiency remains approximately within 51.43–76.23%. The small differences and local variations between simulated and measured results are mainly attributed to practical measurement effects, including connector and cable losses, chamber alignment, fabrication tolerance, substrate variation, and calibration uncertainty.

**Figure 21:**
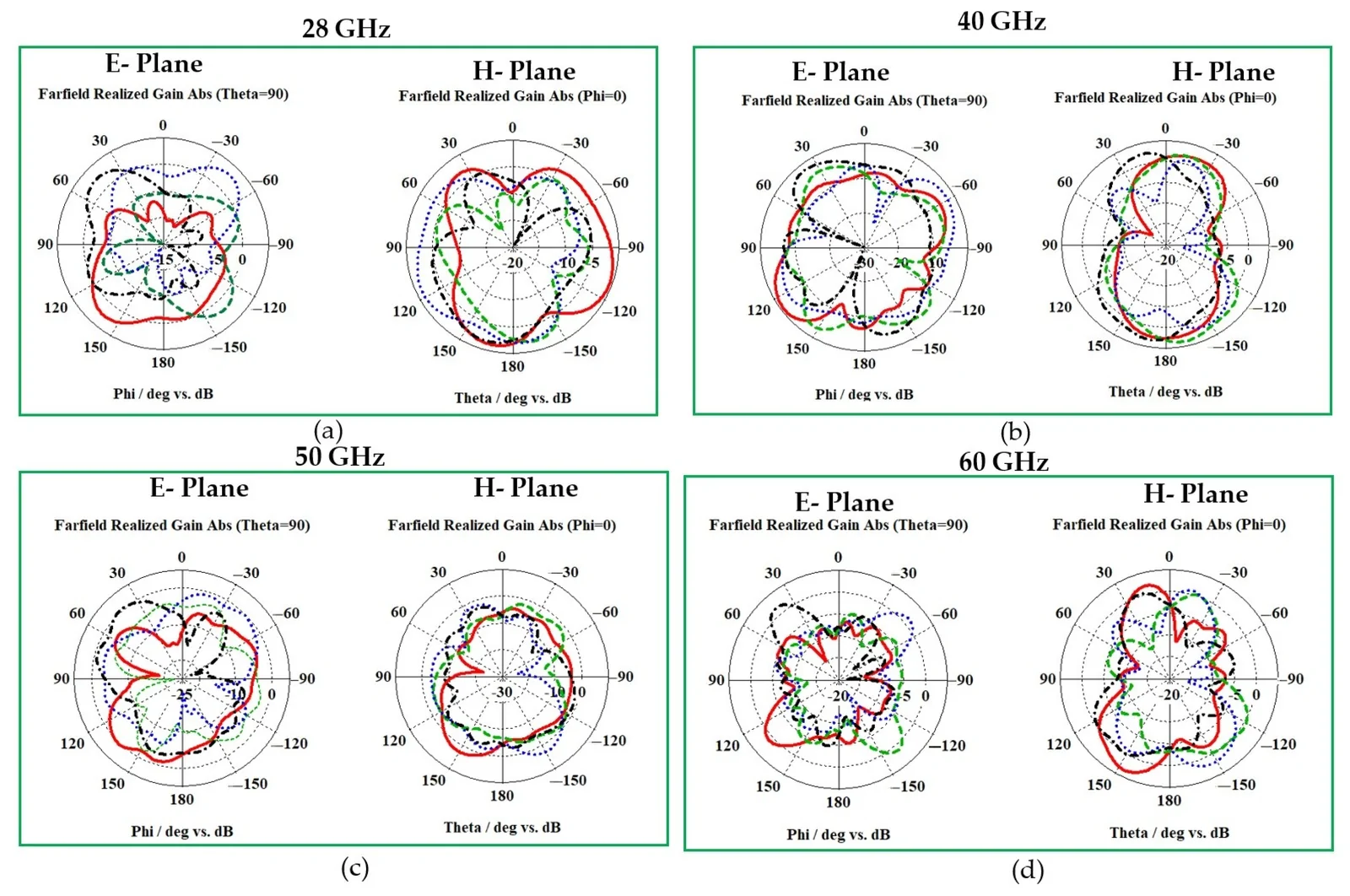
Simulated Radiation patterns at all ports in Free space for the proposed HSMA at (**a**) 28 GHz, (**b**) 40 GHz, (**c**) 50 GHz and (**d**) 60 GHz.

Before presenting the measured radiation-pattern results, the simulated free-space radiation patterns of all four HSMA ports are analyzed to clarify the port-dependent radiation behavior of the proposed MIMO configuration. Figure 21 shows the realized-gain polar patterns in the E-plane and H-plane at representative frequencies of 28, 40, 50, and 60 GHz. For each frequency, the four ports are excited individually while the remaining ports are terminated with matched loads.

The results show that all ports maintain usable radiation behavior across the selected frequencies, but their pattern shapes and main radiation directions are not identical. This difference is expected because the four Hilbert-slot elements are positioned at different orientations within the compact MIMO layout. As a result, each port interacts differently with the surrounding structure, feeding position, and neighboring elements. The observed port-dependent radiation behavior is beneficial for MIMO operation because it provides pattern diversity and allows different ports to support different propagation directions.

At lower and mid-band frequencies, such as 28 and 40 GHz, the radiation patterns remain relatively broad, which is useful for maintaining coverage in short-range wearable communication. At higher frequencies, such as 50 and 60 GHz, the patterns become more directional and show stronger port-to-port variation. This behavior is consistent with the electrically larger size of the antenna at higher mmWave frequencies and the stronger influence of element orientation on the radiated field.

These simulated free-space results justify the use of selected representative ports in the subsequent measured and on-body analyses. Since each port radiates differently, the measured radiation-pattern study is carried out using the port most relevant to the intended measurement or telemetry direction, while the simulated all-port results demonstrate that the four-port HSMA provides additional radiation diversity. This supports the suitability of the proposed antenna for reliable mmWave wearable telemetry, where alternative ports can be used to improve link robustness under orientation changes, body shadowing, or local detuning.

The simulated and measured 2-D polar radiation patterns of the proposed four-port HSMA are shown in Figure 21 for Port 1 at 28, 40, 50, and 60 GHz. The patterns are presented in the E-plane and H-plane to evaluate the practical radiation behavior of the fabricated prototype under free-space measurement conditions. Port 1 is selected as the measured representative port, while the simulated all-port radiation patterns discussed earlier demonstrate the port-dependent radiation behavior of the four-port MIMO configuration.

**Figure 22:**
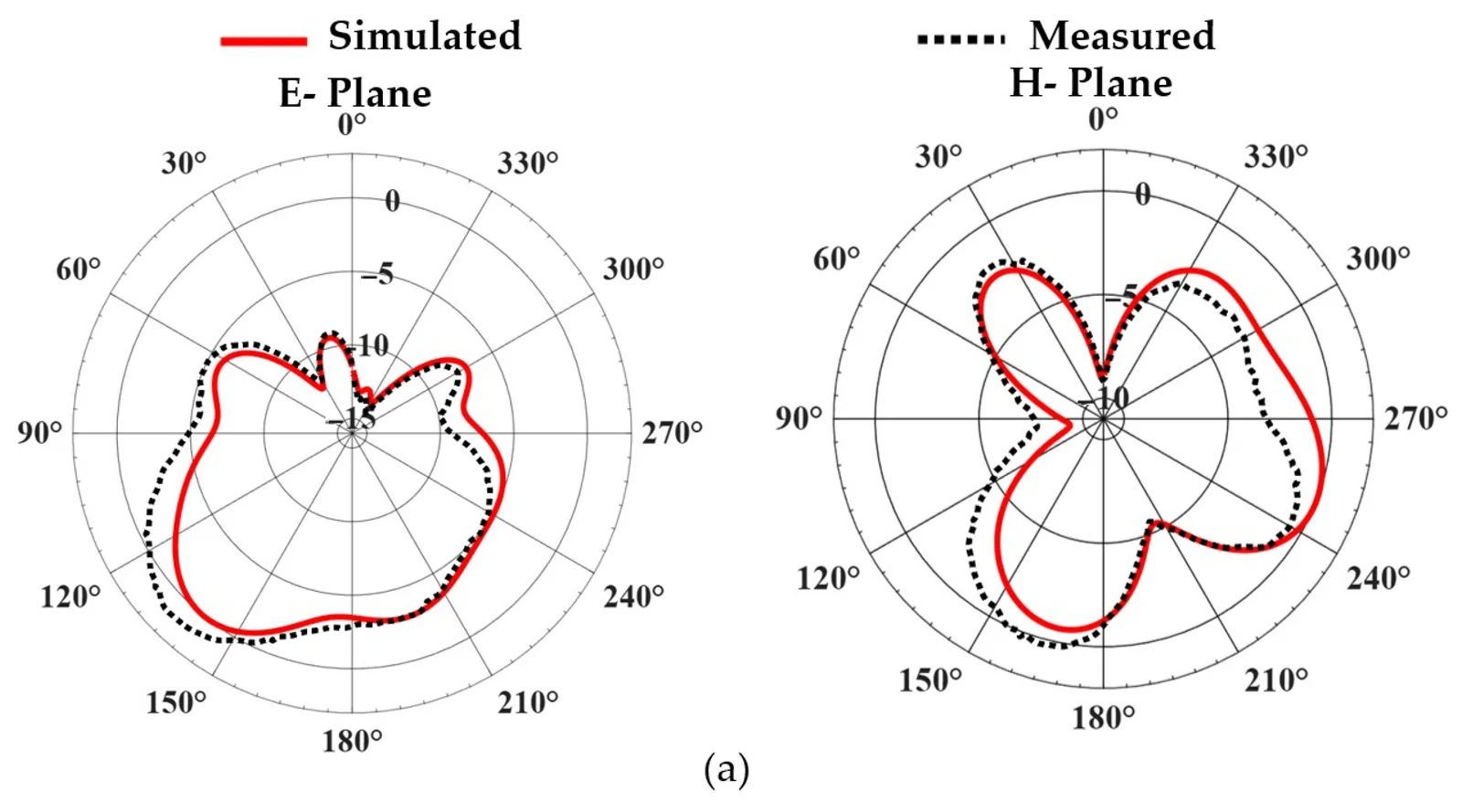

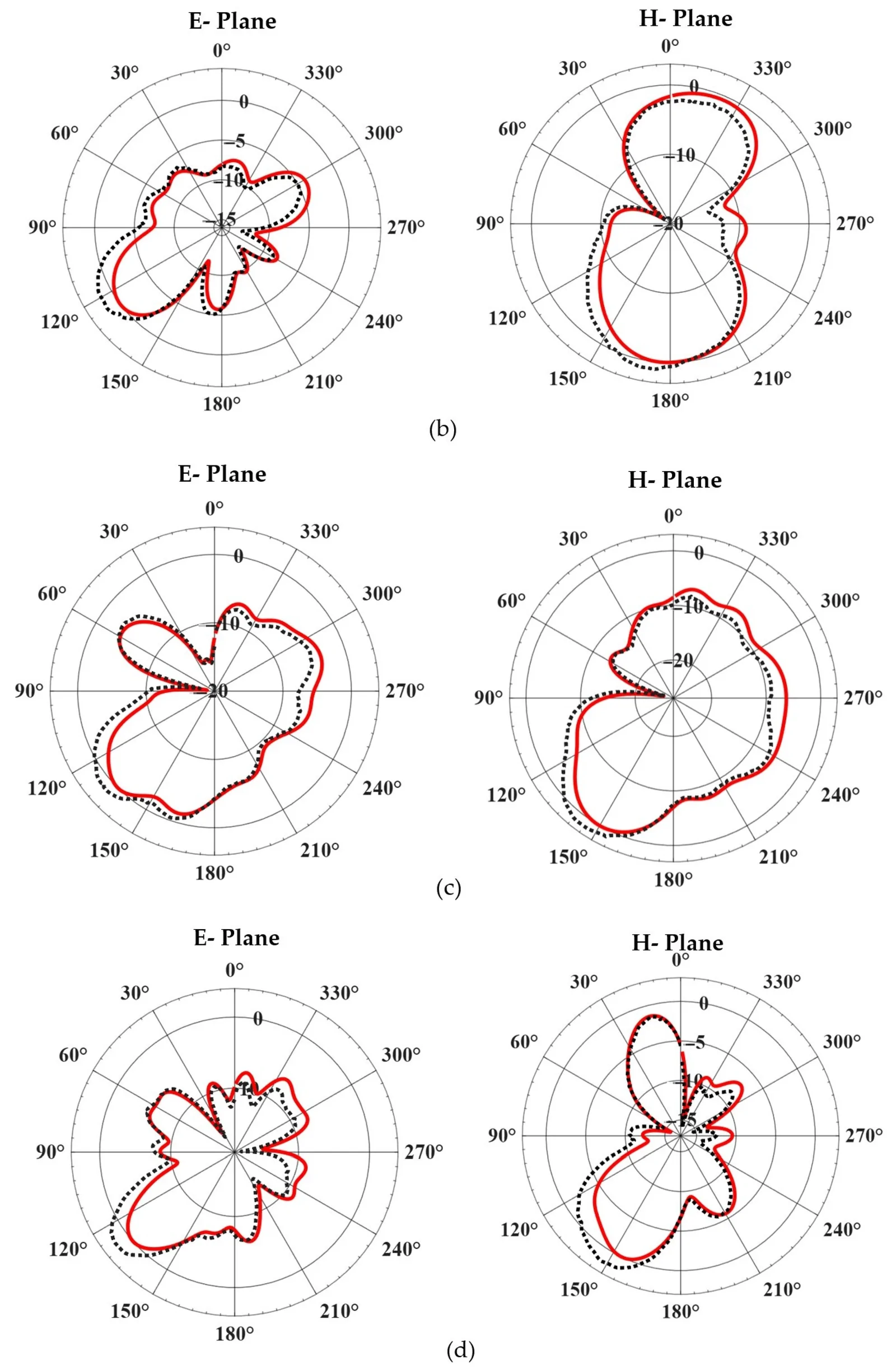
Simulated and measured radiation pattern of the proposed compact HSMA at (**a**) 28 GHz, (**b**) 40 GHz, (**c**) 50 GHz and (**d**) 60 GHz.

As shown in Figure 22, the measured patterns generally follow the simulated responses at all frequencies investigated. At 25 GHz, the measured and simulated patterns show similar broad radiation behavior, confirming that the fabricated HSMA maintains stable low-band radiation performance. At 40 GHz, the measured pattern preserves the main radiation shape observed in simulation, with only moderate deviation in some angular regions. At 50 GHz and 60 GHz, stronger pattern variations are observed, which is expected at higher mmWave frequencies because small fabrication tolerances, connector effects, cable movement, probe alignment, and chamber positioning can have a greater influence on the measured field distribution.

Despite these differences, the measured results confirm that the fabricated HSMA maintains practical radiation characteristics across the selected mmWave frequencies. The agreement between simulated and measured patterns verifies the validity of the proposed design and confirms that Port 1 can provide stable radiation behavior in free space. Together with the simulated all-port radiation-pattern analysis, these measured results support the suitability of the proposed four-port HSMA for mmWave MIMO operation and wearable telemetry applications.

The discrepancy between the simulated and measured results can be attributed to several factors, including fabrication tolerances, variations in measurement setup, and differences in material properties. Minor inaccuracies in the manufacturing process, equipment calibration, or antenna positioning during testing can cause deviations. Additionally, the assumption of ideal materials in simulations may not perfectly match real-world conditions. Despite these factors, the overall performance trends, including gain and radiation efficiency, show good agreement, confirming the antenna’s practical feasibility for the intended applications.

### MIMO Diversity Parameters

To further evaluate the MIMO performance of the proposed four-port HSMA, the diversity parameters are analyzed in terms of envelope correlation coefficient (ECC) and diversity gain (DG). These parameters provide a more complete assessment of the independence between the antenna ports, the diversity improvement, the received-power balance, the channel-capacity degradation, and the active reflection behavior of the multi-port antenna. In MIMO antenna systems, low correlation between antenna ports is required to support independent signal paths and improve communication reliability. The ECC is therefore an important metric because it indicates the correlation between the signals received by different antenna ports. Low ECC values indicate weak correlation and better MIMO diversity behavior.

For the proposed four-port HSMA, the ECC is evaluated for the six possible port pairs, namely ECC_12_, ECC_13_, ECC_14_, ECC_23_, ECC_24_, and ECC_34_. For a four-port MIMO antenna, the S-parameter-based ECC between ports i and j can be calculated as

(7) $$ECC_{ij}=\frac{=\sum_{n=1}^{4}{\left|S_{in}^{*}S_{jn}\right|}^{2}}{\left(1-\sum_{n=1}^{4}{\left|S_{in}\right|}^{2}\right)\left(1-\sum_{n=1}^{4}{\left|S_{jn}\right|}^{2}\right)}$$

where i and j denote the selected antenna ports, n is the summation index over the four ports, and * denotes the complex conjugate. A lower ECC indicates lower correlation between the ports and better independence of the received signals. In practical MIMO systems, ECC values below 0.5 are generally regarded as acceptable, while values close to zero indicate excellent diversity behavior.

The diversity gain is calculated from the ECC as

(8) $$DG_{ij}=10\sqrt{1-{\left|ECC_{ij}\right|}^{2}}$$

where $DG_{ij}$ represents the diversity gain between ports i and j. A DG value close to 10 dB indicates near-ideal diversity performance. Therefore, low ECC together with DG close to 10 dB confirms that the antenna ports can provide independent radiation paths and improved link reliability.

**Figure 23:**
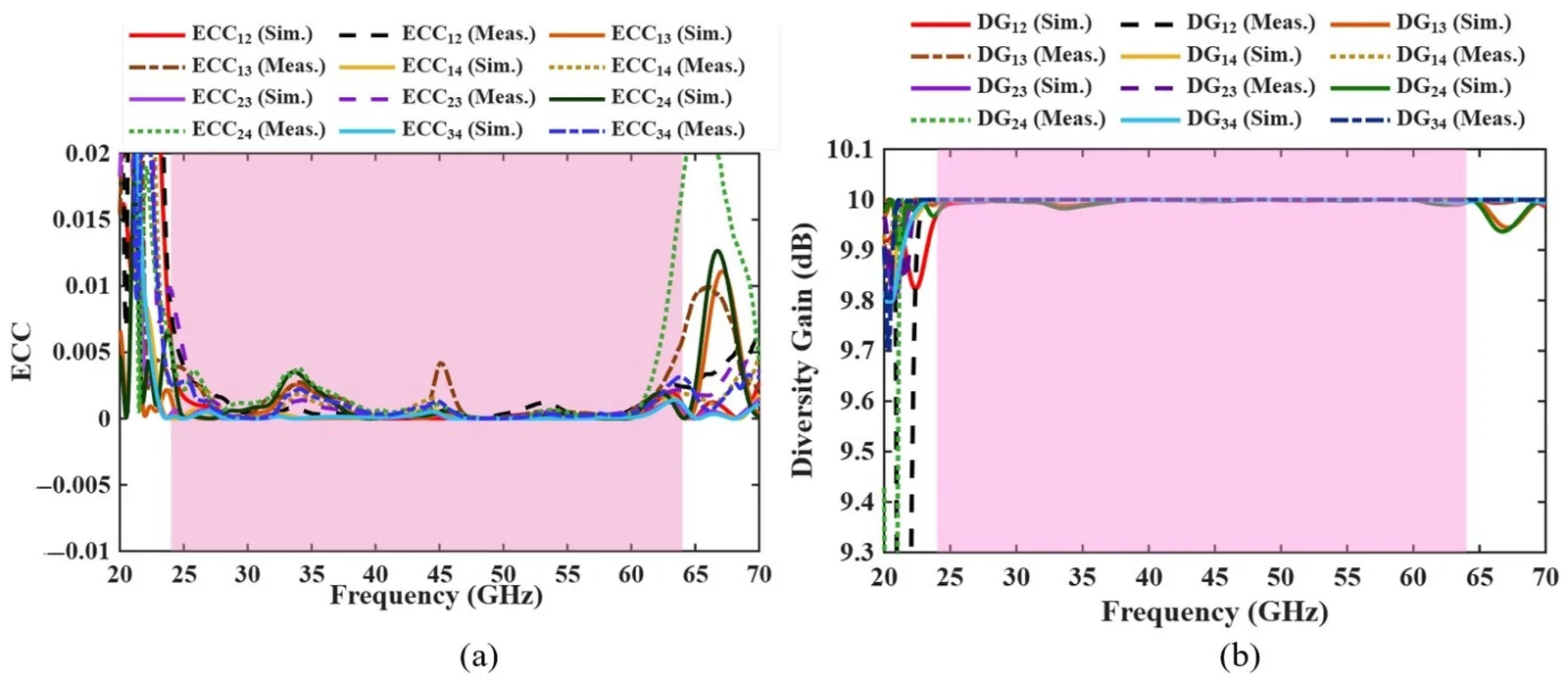
Simulated and measured (**a**) ECC and (**b**) DG of the proposed HSMA.

Figure 23 presents the simulated and measured MIMO diversity performance of the proposed four-port HSMA in terms of ECC and DG over the highlighted 24–64 GHz operating band. As shown in Figure 23a, the ECC values for all investigated port pairs, including ECC_12_, ECC_13_, ECC_14_, ECC_23_, ECC_24_, and ECC_34_, remain very close to zero across most of the operating band. The simulated and measured ECC responses show similar trends, with only small variations observed near some frequency regions. From the plotted results, the ECC values remain well below 0.02 within the required band, which is much lower than the commonly accepted limit of 0.5 for practical MIMO systems. This confirms weak correlation between the antenna ports and indicates that the proposed four-port configuration can provide independent signal paths.

The low ECC behavior agrees with the S-parameter results discussed earlier, where the isolation remains mostly below −20 dB across the main operating band. This confirms that the closely spaced ports maintain good isolation and limited mutual coupling. The small differences between simulated and measured ECC responses are expected and can be attributed to fabrication tolerances, connector effects, measurement uncertainty, and the slight variations observed in the measured matching and transmission coefficients.

Figure 23b shows the corresponding simulated and measured DG responses. The DG values remain close to the ideal value of 10 dB across the highlighted 24–64 GHz band for all port combinations. Although small deviations appear near the lower-frequency edge and outside the main operating band, the DG remains highly stable within the required band, confirming effective diversity performance. Since DG is directly related to ECC, the near-10 dB response further verifies the low correlation and strong diversity capability of the proposed HSMA. The combination of wide impedance bandwidth, mutual coupling mostly better than 20 dB, ECC values close to zero, and DG values close to 10 dB demonstrates that the proposed antenna can support independent communication paths and help reduce the effects of fading, body shadowing, posture variation, and orientation-dependent propagation in body-centric communication scenarios.

As shown in Table 3, the reported MIMO antennas provide useful performance in terms of isolation, gain, ECC, DG, and wearable validation. However, most existing designs are limited to dual-band, tri-band, multiband, or limited wideband operation. For example, several reported antennas operate only around selected 28/38 GHz bands, sub-6 GHz bands, or partial mmWave ranges, while others require larger antenna footprints, defected ground structures, fractal decoupling features, or modified shared-ground arrangements to achieve acceptable isolation. In contrast, the proposed HSMA provides continuous super-wideband operation from 24.05 to 62.64 GHz within a compact 12.32 × 12.32 × 0.508 mm^3^ footprint, making it one of the most compact four-port MIMO designs in the comparison.

The significance of the proposed design is further highlighted by its ability to combine compactness, wide bandwidth, and MIMO diversity without relying on a separate complex decoupling network. The Hilbert-slot compact layout increases the effective current path within a limited physical area, while the 2 × 2 port arrangement, optimized gap, feed, and ground dimensions help maintain inter-port isolation mostly better than 20 dB. In addition, the proposed antenna achieves a measured peak realized gain of 7.52 dBi, ECC below 0.017, and DG of approximately 9.9985 dB, confirming low correlation and effective diversity performance across the required mmWave band.

Compared with the listed wearable and mmWave MIMO antennas, the proposed HSMA also provides a more complete validation route for wearable biomedical telemetry. While some previous works include SAR only, bending only, or antenna-level MIMO parameters, the proposed work considers SAR, bending performance, and CST-simulated link performance. Therefore, the proposed HSMA does not only demonstrate strong antenna-level performance but also evaluates its practical suitability for body-centric and wearable telemetry communication. This combination of compact size, continuous super-wideband operation, low mutual coupling, strong diversity performance, and wearable link validation makes the proposed HSMA a promising candidate for 5G FR2 wearable biomedical telemetry, WBAN, and future body-centric mmWave communication systems.

## 4. Conclusions

This work presented a compact super-wideband four-port Hilbert-slot MIMO antenna for 5G FR2 mmWave wearable medical telemetry and body-centric communication. The proposed HSMA was designed on a semi-flexible Rogers RT/Duroid 5880 substrate with a compact overall footprint of 12.32 × 12.32 × 0.508 mm^3^, while maintaining wide impedance matching across the required mmWave operating band. The fabricated prototype confirmed good agreement with the simulated results, with the representative measured S_11_ response remaining below −11.86 dB from 24.71 to 63.81 GHz. The measured and simulated inter-port transmission coefficients also remained mostly below −20 dB over the main operating band, confirming good isolation between the closely spaced MIMO ports. In addition, the antenna maintained useful realized gain and radiation efficiency across the highlighted 24–64 GHz band, with measured responses following the simulated trends despite expected practical deviations caused by fabrication tolerance, connector/cable effects, substrate variation, and measurement uncertainty.

The MIMO diversity performance was further verified using ECC and DG. The ECC values for all four port pairs remained close to zero and well below the accepted 0.5 limit, while the DG values remained close to 10 dB across the required band. These results confirm that the proposed four-port HSMA can provide weakly correlated and independent communication paths, which is essential for improving link reliability in wearable medical telemetry scenarios affected by body shadowing, posture variation, and orientation-dependent propagation. The antenna was also evaluated under bending and realistic body-loading conditions using layered and CST Gustav voxel phantoms, where it maintained practical impedance, isolation, and radiation behavior. The SAR analysis confirmed the electromagnetic safety of the proposed wearable configuration, while the CST-simulated S-parameter-based link analysis demonstrated the feasibility of selected body-centric telemetry paths through received-power and link-margin evaluation. Overall, the proposed compact HSMA combines super-wideband operation, strong MIMO isolation, favorable diversity performance, body-compatible operation, and link-level validation, making it a promising candidate for next-generation mmWave wearable medical monitoring and body-centric communication systems.

## Figures and Tables

**Table 1:** Parametric study of the proposed four-port HSMA.

Par.	Investigated Values (mm)	Optimized (mm)	Effect on $S_{11}$ and Gain	Effect on Isolation
$L_{l}$	0.18, 0.20, 0.22	0.18	Controls Hilbert current path and band position.	Maintains balanced isolation.
$L_{f}$	0.8, 1.0, 1.2	1.0	Tunes feed coupling and matching.	Reduces feed-induced coupling.
$W_{f}$	1.45, 1.75, 2.05	1.75	Adjusts feed impedance and gain stability.	Improves isolation stability.
$L_{m}$	0.9, 1.0, 1.1	1.0	Smooths the feed-to-slot transition.	Helps reduce port coupling.
$W_{m}$	1.4, 1.5, 1.6	1.5	Balances matching bandwidth and gain.	Supports stable isolation
$L_{sl}$	0.8, 0.95, 1.1	0.95	Controls slot loading and resonance spread.	Limits unwanted current coupling.
$W_{sl}$	0.8, 1.0, 1.2	1.0	Tunes slot coupling and impedance response.	Preserves acceptable isolation.
extl	0.8, 1, 1.2	1.0	Extends current path and improves matching.	Stabilizes adjacent-port coupling.
extw	0.6, 0.8, 1.0	0.8	Adjusts extension width for gain/matching balance.	Avoids excessive radiating-region coupling.
Subx	12.32, 12.82, 13.32	12.32	Preserves compactness with stable response.	Maintains compact MIMO isolation.
Suby	12.32, 12.82, 13.32	12.32	Preserves compactness without degrading matching.	Avoids unnecessary coupling increase.
Gndy	1.7, 1.9, 2.1	1.9	Tunes ground effect and impedance response.	Gives balanced coupling behavior.

**Table 2:** Simulated 2D and 3D radiation patterns of the proposed four-port HSMA on the CST Gustav voxel phantom for the telemetry-oriented active ports: Port 4 (knee), Ports 2 and 3 (wrist), and Port 1 (shoulder).

Freq. (GHz)	Knee (Port 4)	Wrist		Shoulder (Port 1)
		(Port 2)	(Port 3)	
28	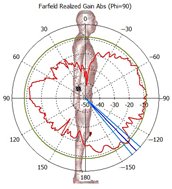	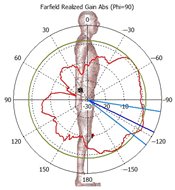	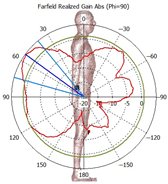	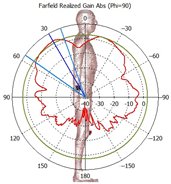
	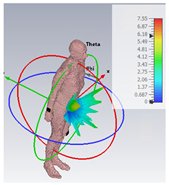	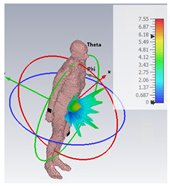	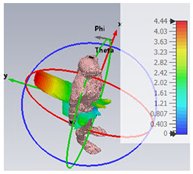	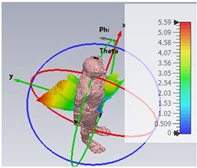
40	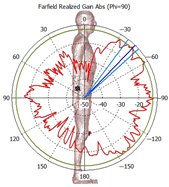	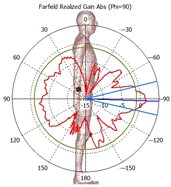	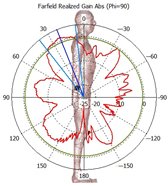	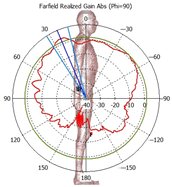
	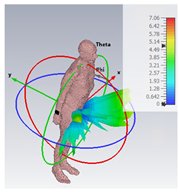	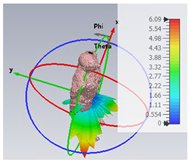	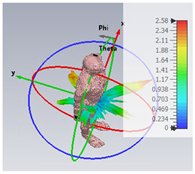	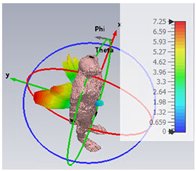
50	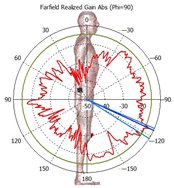	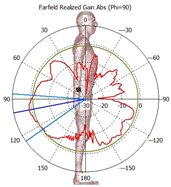	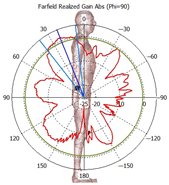	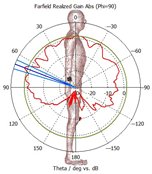
	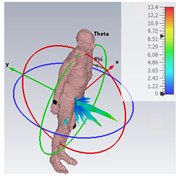	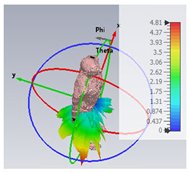	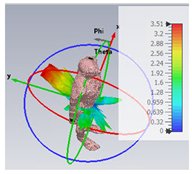	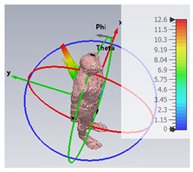
60	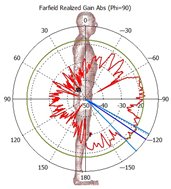	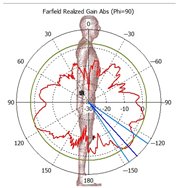	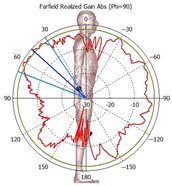	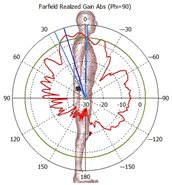
	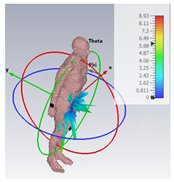	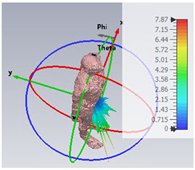	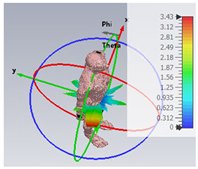	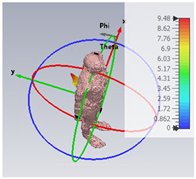

**Table 3:** Comparison with existing mmWave 5G MIMO wearable antennas in the literature.

Ref.	Configuration	Volume (mm × mm × mm)	Operating Band (GHz)	Isolation (dB)	Gain (dBi)	ECC; DG (dB)	Compactness/ Isolation Technique	Application	Wearable Validation
[6]	2 × 2 (4-port)	18 × 8.5 × 0.25	27.76–28.48/37.69–38.19	>20	7.73	<0.03; 9.89	DGS	5G cellular	SAR only
[7]	1 × 4 (4-port)	44 × 11 × 0.25	26.28–27.36, 27.94–28.62, 32.33–33.08, and 37.59–39.47 GHz	>23.2	8.72	<0.02; 9.97	Flexible dual-band structure	Wearable 5G	SAR, Bending
[9]	2 × 2 (4-port)	40 × 30 × 1.67	2–3/3.4–3.9/4.4–5.2	>20	6.2	Acceptable, NA	Sierpiński fractal + Hilbert CRLH	Sub-6 GHz 5G	No
[10]	2 × 2 (4-port)	10.6 × 10.6 × 0.2	15.97–45.12	>20	NA	<0.007; >9.965	Cross fractal + DSSR	K/Ka-band 5G	No
[12]	1 × 2 (2-port)	45 × 75 × 1.6	1–21.4/23.9–30	High	~9.0	<0.02; >9.99	Koch-Minkowski fractal	5G/UWB	No
[14]	1 × 2 (2-port)	28.22 × 44 × 1.6	17.7–28	>31.4, 22.8, 26.8, 22, 24.4	8.46	0.04; >9.99	Spherical fractal + DGS	5G NR n258	No
[15]	2 × 2 (4-port)	30 × 12 × 0.508	25.52–28.23/36.5–37.5/47.7–49.7	>12, 38 and 33	11	NA; NA	Wide-beam planar antenna	Wearable off-body	SAR only
[16]	2 × 2, 4-port	30 × 30 × 0.8	26.5–41	≈30	4–6	<0.003; ≈10	Koch fractal miniaturization + modified shared ground plane	5G NR/6G mmWave	Bending only
[17]	2 × 2/4 × 4	55 × 110 × 1.6 /110 × 110 × 1.6	5.1, 8.7, 10.3, 10.6, 13.8, 18.3, 20.7, 22.3	>37	up to 6.7	≤0.0027; >9.99	Natural cotton fabric + Lorentz-shaped elliptical fractal; high isolation without DGS	Wearable 5G/advanced wireless/military defense	Fabricated prototype and natural-fabric flexibility; no SAR/bending study reported
Proposed	2 × 2 (4-port)	12.32 × 12.32 × 0.508	24.05–62.64	≥20	7.52	<0.017; 9.9985	Hilbert-slot compact layout; isolation by port orientation and optimized gap/feed/ground.	Wearable biomedical telemetry/WBAN/5G	SAR, Bending, Link Performance

## Data Availability

The original contributions presented in this study are included in the article. Further inquiries can be directed to the corresponding author.
